# Negative and zero linear compressibility in copper dicyanamide and tricyanomethanide

**DOI:** 10.1039/d5sc04854k

**Published:** 2025-12-17

**Authors:** Muzi Chen, Hanna L. B. Boström, Dominik Daisenberger, Nicholas P. Funnell, Christopher J. Ridley, Andrew B. Cairns

**Affiliations:** a Department of Materials, Imperial College London, Royal School of Mines Exhibition Road SW7 2AZ London UK a.cairns@imperial.ac.uk muzichen@kofo.mpg.de Tel: +44(0)20 7594 9528; b London Centre for Nanotechnology, Imperial College London London SW7 2AZ UK; c Department of Chemistry, Stockholm University Svante Arrhenius väg 16C SE-106 91 Stockholm Sweden; d Wallenberg Initiative Materials Science for Sustainability, Department of Chemistry, Stockholm University SE-114 18 Stockholm Sweden; e Diamond Light Source Ltd Harwell Campus Didcot OX11 0DE UK; f ISIS Neutron and Muon Source, Rutherford Appleton Laboratory Harwell Campus Didcot OX11 0QX UK

## Abstract

Rutile-structured materials can exhibit negative linear compressibility (NLC) following ferroelastic phase transitions, expanding in one direction under uniform compression. We investigate this phenomenon in structural analogues—transition metal dicyanamides (dca) and tricyanomethanides (tcm) with single and double rutile-like structures, respectively. The pressure-induced structural behaviour of Cu(tcm)_2_ and Cu(dca)_2_ are studied using high-pressure diffraction. Both systems undergo anisotropic deformation upon compression, with Cu(dca)_2_ exhibiting NLC of −6.5(10) TPa^−1^ along the *c*-axis, while Cu(tcm)_2_ shows zero linear compressibility (ZLC) along the *a*-axis. This difference is attributed to the single rutile-like network with flexible dca^−^ linkers in Cu(dca)_2_, in contrast to the more constrained doubly interpenetrating structure of Cu(tcm)_2_ with rigid tcm^−^ linkers. We also study the interplay between structural features and electronic effects arising from the Jahn–Teller distortion in both materials, in controlling their compression behaviour.

## Abstract 2

Single *vs.* double networks: Cu(dca)_2_ shows NLC = −6.5 TPa^−^^1^*via* flexible dca^−^ then transitions to monoclinic at 1.11 GPa; Cu(tcm)_2_ exhibits ZLC through rigid tcm^−^ maintaining structure to 3.75 GPa.

## Graphical abstract

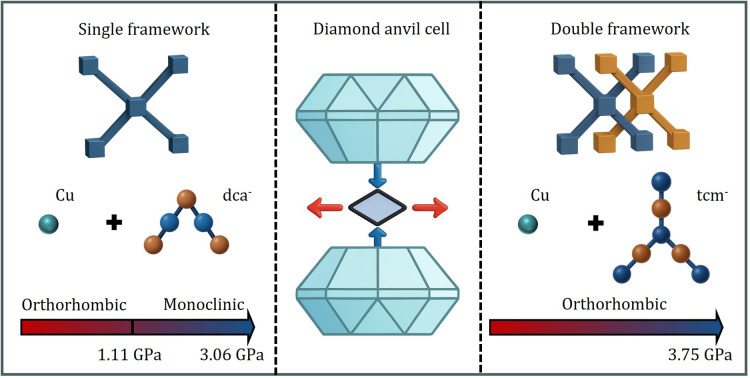

## 1 Introduction

Negative linear compressibility (NLC) is the property of materials to increase in length along one direction when hydrostatically compressed.^1^ This has attracted interest for potential applications in various fields, such as molecular-scale artificial muscles and pressure sensors.^2^ To date, NLC has been identified in a wide variety of materials, including inorganic oxides, molecular solids, metal cyanides, and metal–organic frameworks (MOFs).^3–7^ The most impressive NLC performances of existing materials are shown by metal cyanides. Their NLC mechanisms are based on the pressure-induced deformation of flexible frameworks. The rapid compression in one direction is transferred to an expansion in the perpendicular direction *via* flexing of the framework. Two typical examples are Zn[Au(CN)_2_]_2_ and Ag_3_[Co(CN)_6_] with flexible “honeycomb” and “wine-rack” frameworks, respectively.^8,9^ Despite these advances, research efforts continue, aiming to uncover novel NLC materials with superior properties—such as larger compressibility magnitudes, broader pressure ranges of operation, or enhanced mechanical stability—or operating through alternative mechanisms. The ultimate goal is to gain a deeper understanding of the underlying principles that govern this exceptional property.

**Fig. 1:**
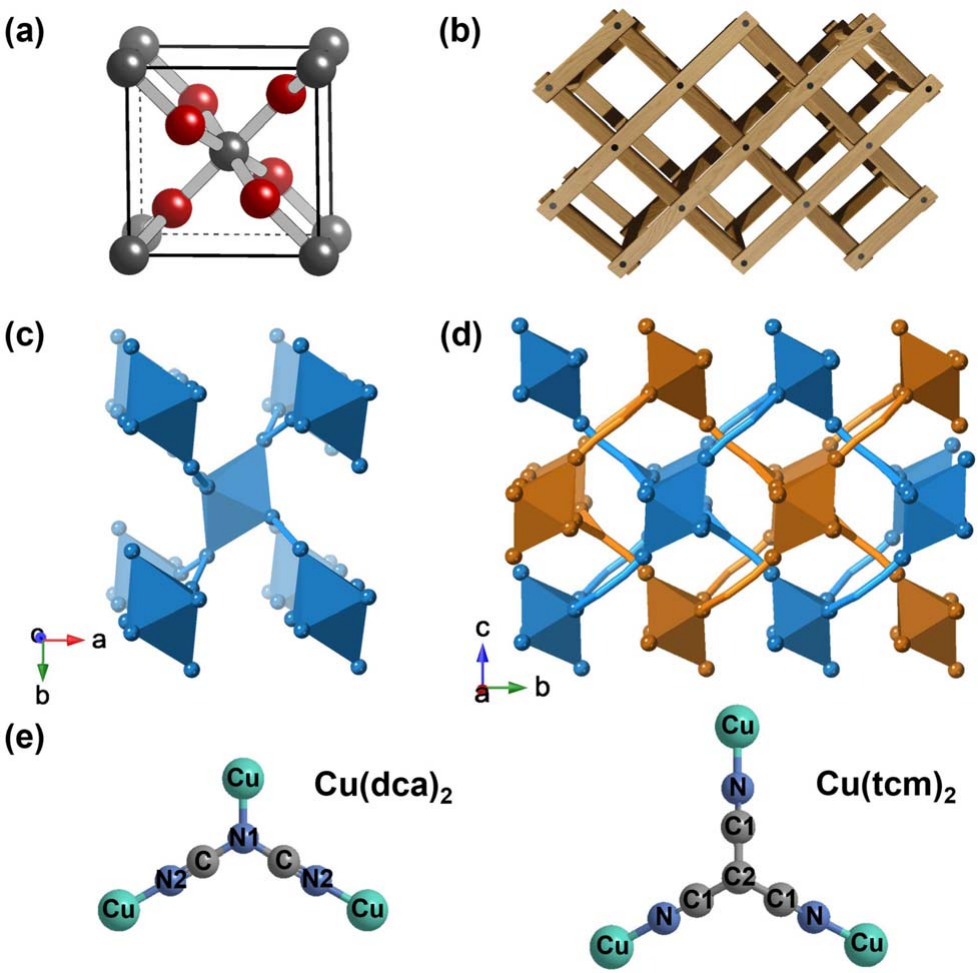
(a) Unit cell of rutile (TiO_2_) structure.^21^ (b) Schematic illustration of the “wine-rack” topology in rutile-structured solids. Crystal structures of (c) Cu(dca)_2_ and (d) Cu(tcm)_2_, viewed along the *c*- and *a*-axes, respectively. The dca^−^ and tcm^−^ ligands are shown as simplified stick representations with carbon atoms omitted (C in Cu(dca)_2_); C1, C2 in Cu(tcm)_2_ to better illustrate the network topology. All octahedra represent CuN_6_ coordination environments. Cu(dca)_2_ has a single rutile-like network, while Cu(tcm)_2_ consists of two interpenetrating rutile-like networks distinguished by different colours. (e) Molecular structures and coordination modes of dca^−^ and tcm^−^ ligands with Cu^2+^ centres.

Rutile, one of the three crystalline polymorphs of titanium dioxide (TiO_2_), has a well-known tetragonal crystal structure composed of columns of edge-sharing TiO_6_ octahedra (as shown in Fig. 1).^10,11^ This structure is ferroelastically unstable at high pressures or low temperatures due to the coordinated rotation of adjacent octahedral columns, resulting in a distorted orthorhombic CaCl_2_-like structure.^12,13^ The main difference between these two polymorphs is the rotation of the edge-sharing octahedra around the *c*-axis, which breaks the 4-fold symmetry in CaCl_2_-like structures.^14,15^ Several dioxides and difluorides with the rutile structure undergo similar phase transitions, with the resulting low-symmetric CaCl_2_-like phases exhibiting NLC in one of the orthogonal directions.^16^ These transitions are directly responsible for inducing NLC in rutile-structured solids.

Furthermore, they are classified as proper ferroelastic transitions, with strain tensor components acting as primary order parameters.^17,18^ This transition is associated with the softening of a B_1g_ mode (ferroelastic instability) involving octahedral rotation within the framework, which spontaneously generates a symmetry-breaking strain.^19^ When volume continuity is maintained during compression, the emergence of spontaneous strain can necessitate lengthening in at least one axial direction to stabilize the lower symmetry phase.^20^ This elongation occurs because the material must compensate for contraction in other directions while preserving its overall volume, resulting in anisotropic deformation. Consequently, above the transition pressure, NLC is observed in such proper ferroelastic materials.

In addition to simple binary inorganic solids, the rutile topology has been reported in a variety of hybrid materials, in which the transition metal octahedra are connected by molecular linkers.^22–24^ Transition metal dicyanamide and tricyanomethanide are two examples, abbreviated M(dca)_2_ and M(tcm)_2_ (M = Mn, Fe, Co, Ni, Cu).^25^ M(dca)_2_ exhibits a single rutile-like network in which the M^2+^ cations are octahedrally coordinated to two dca^−^ ligands *via* the nitrile and amide nitrogen donor atoms. In contrast, M(tcm)_2_ exhibits a doubly interpenetrating structure with bonds exceeding 4.5 Å between the octahedral nodes (see Fig. 1(e)).^25^ These materials preferentially adopt CaCl_2_-type orthorhombic structures rather than rutile-type tetragonal structures at ambient conditions. Unlike rutile-structured dioxides and difluorides, M(dca)_2_ and M(tcm)_2_ do not undergo ferroelastic rutile-CaCl_2_-type phase transitions under pressure.

However, previous studies suggest that these materials still exhibit NLC behaviour when subjected to external pressure.^26,27^ A notable example is Co(dca)_2_, which exhibits NLC with a value of −3.7(3) TPa^−1^ over the pressure range of 0.05–1.11 GPa.^27^ This NLC value is higher than those observed for binary inorganic solids with rutile topology—for instance, MgF_2_ with *K*_NLC_ = −1.3(3) TPa^−1^ and MnO_2_ with *K*_NLC_ = −0.16(7) TPa^−1^.^1,16^ This indicates that flexible molecular linkers enhance the responsiveness to external mechanical stimuli, leading to more pronounced structural changes.^28^ Within the family of transition metal dicyanamides and tricyanomethanides, variations in the metal centres can significantly alter their mechanical properties. Factors such as ionic radius, electronic configuration, and coordination preferences can all influence the framework flexibility and thus the material's response to pressure.^29^ An investigation of the relationship between chemical composition, crystal structure, and NLC properties within these materials is therefore particularly important for developing design principles for NLC materials.

This paper focuses on Cu(dca)_2_ and Cu(tcm)_2_. These materials introduce additional structural complexity due to the Jahn–Teller distortion of their octahedral metal sites. Jahn–Teller distortion is a phenomenon that allows a degenerate electronic state to be deformed into a system of reduced symmetry. This transformation eliminates electronic degeneracy, thereby reducing energy levels.^30^ Notably, a pronounced Jahn–Teller effect manifests in d^9^ octahedral complexes, such as Cu(ii).^31^ This d^9^ electronic configuration populates three electrons into two e_g_ orbitals, resulting in a doubly degenerate ground state. To eliminate orbital and electronic degeneracy, Cu(ii) complexes are deformed along the *z*-axis. However, the Jahn–Teller theorem does not dictate the direction of the distortion, but merely predicts the presence of an unstable geometry. Typically, the bonds to the ligands undergo stretching along the *z*-axis, although in certain cases they may instead shorten.^32^

For Cu(dca)_2_ and Cu(tcm)_2_, a significant elongation of the Cu–N bond along the *z*-axis is observed. These elongated bonds, due to the Jahn–Teller effect, show a pronounced sensitivity to pressure. They are prone to rapid reduction upon compression.^33^ Evidence for this phenomenon has been found in a number of coordination complexes with Cu^2+^. For example, Cu[Pt(CN)_6_] shows a higher compressibility along the *c*-axis (*K*_c_ = 15.3(2) TPa^−1^) compared to the *a*-axis (*K*_*a*_ = 4.82(11) TPa^−1^). This difference is attributed to the Jahn–Teller distorted bonds present along the *c*-axis.^34,35^ With this in mind, we hypothesize that Jahn–Teller distortions could play a crucial role in enhancing the NLC properties of materials. The rapid compression in certain directions could be converted into substantial NLC behaviour in the perpendicular direction *via* the hinge mechanism in wine-rack framework.

Here, the pressure-induced structural changes of Cu(dca)_2_ and Cu(tcm)_2_ are studied using high-pressure powder X-ray diffraction (HP-PXRD) and high-pressure powder neutron diffraction (HP-PNRD), respectively. The results reveal a remarkable NLC of −6.5(10) TPa^−1^ along the *c*-axis over the pressure range of 0.05–1.11 GPa in the orthorhombic phase of Cu(dca)_2_ (Cu(dca)_2_-I). Above 1.11 GPa, Cu(dca)_2_ undergoes a phase transition from orthorhombic to monoclinic. It remains in the monoclinic phase, Cu(dca)_2_-II, in the pressure range 1.11–3.06 GPa and shows no NLC behaviour. In contrast to Cu(dca)_2_, Cu(tcm)_2_ maintains its orthorhombic structure over a wide pressure range of 0.05–3.75 GPa without undergoing any phase transition. It exhibits zero linear compressibility (ZLC) in the *a*-axis. Through an in-depth analysis of Cu(dca)_2_ and Cu(tcm)_2_, and a comparative study with other rutile-like frameworks, we aim to elucidate the influence of three key factors on the magnitude of their NLC. These factors are (i) the framework topology, (ii) the unit cell density, and (iii) the electronic state of the metal cations. The interplay between these variables determines the magnitude of NLC in materials with rutile topology.

## 2 Experimental

### 2.1 Synthesis

Anhydrous Cu(dca)_2_ was synthesised according to the literature described synthesis procedure for Mn(dca)_2_.^36^ An 8 mL aqueous solution of Na(dca) (178.1 mg, 2 mmol) was added to a 2 mL aqueous solution of Cu(NO_3_)_2_·3H_2_O (241.6 mg, 1 mmol) with vigorous stirring, resulting in immediate precipitation of green-blue microcrystalline powder. To ensure complete reaction, the mixture was stirred for five minutes. The product was collected by filtration, washed with small amounts of deionized water and ethanol to remove impurities, and initially dried at 60 °C overnight. The powder was then heated under vacuum at 100 °C for 5 hours to yield anhydrous Cu(dca)_2_.

Cu(tcm)_2_ was synthesized according to the literature procedure.^37^ A hot solution of K(tcm) (129.2 mg, 1 mmol) in water (4 mL) was added to a hot solution of Cu(NO_3_)_2_·3H_2_O (120.8 mg, 0.5 mmol) in water (3 mL). The solution was then allowed to cool while stirring continuously. A dark brown precipitate appeared some time after the solution reached room temperature. It was filtered, washed with deionized water and dried in an oven overnight (60 °C, 1 day).

### 2.2 Structure characterization

High-pressure powder X-ray diffraction (HP-PXRD) data of Cu(dca)_2_ were collected at beamline I15, Diamond Light Source, with an incident wavelength of 0.4246 Å (100 × 100 µm). A finely ground sample of Cu(dca)_2_ was loaded into a 500 µm hole of a steel gasket in a diamond anvil cell (DAC) with Daphne 7373 as the pressure transmitting medium (PTM) and a ruby sphere for pressure calibration. Pressure was determined by ruby fluorescence on-line at the beginning of each sequence using a Horiba iHR320 spectrometer (473 nm laser). The shift of the R1 fluorescence line of the ruby sphere was measured before and after each data collection, from which the experimental pressure could be calculated. At low pressures (<1 GPa), the error of such measurements is small (typically 0.01 GPa) due to the minimisation of laser power and exposure time; at higher pressures (>1 GPa), the standard error is usually assumed to be 0.1 GPa. Cu(dca)_2_ was measured at pressures ranging from 0.05 to 3.06 GPa. Diffraction images were collected with a Pilatus3 X CdTe 2 M detector located approximately 230 mm from the sample and then integrated and corrected using DAWN software.^38,39^ Lattice parameters and structural models were fitted to the data using Rietveld refinement in the TOPAS software.^40,41^

For Cu(tcm)_2_, HP-PXRD data were collected using a similar strategy at beamline ID27 at the European Synchrotron Radiation Facility (ESRF) over a pressure range of 0.02–0.86 GPa. Hydrostatic pressure was applied using a BETSA membrane diamond anvil cell (DAC) equipped with 600 µm Almax EasyLab type Ia Boehler-Almax design diamonds. A small amount (50 × 50 µm) of powder sample was loaded into the 300 µm diameter hole of a pre-impregnated stainless steel gasket. A small ruby sphere was also placed in the hole for pressure calibration using the ruby fluorescence method described above with silicon oil as the PTM. A wavelength of 0.3738 Å was used (beamsize 80 × 80 µm). Data were collected using a MARCCD detector with an approximate sample-to-detector distance of 306 mm, as calibrated using a CeO_2_ standard. Integration of 2D powder diffraction data was performed using DIOPTAS v 2.4. Calibration, masking, and integration were consistent across all data sets.^42^

High-pressure powder neutron diffraction (HP-PNRD) data of Cu(tcm)_2_ were collected on the PEARL instrument at the ISIS Neutron and Muon Source with a time-of-flight (ToF) transverse detector bank consisting of 9 modules and capable of observing *d*-spacings in the range 0.5–4.1 Å with a nominal average resolution of about 0.64%.^43^ The Cu(tcm)_2_ powder sample was sealed in a zero scattering Ti–Zr gasket together with a Pb pressure marker and a mixture of pentane/isopentane as a pressure transmitting medium. The assembly was then loaded into zirconia-toughened alumina (ZTA) anvils and compression was applied using a Paris-Edinburgh (P-E) press. Measurements were made at pressures ranging from 0.05 to 3.75 GPa at room temperature.

## 3 Results and discussion

**Fig. 2:**
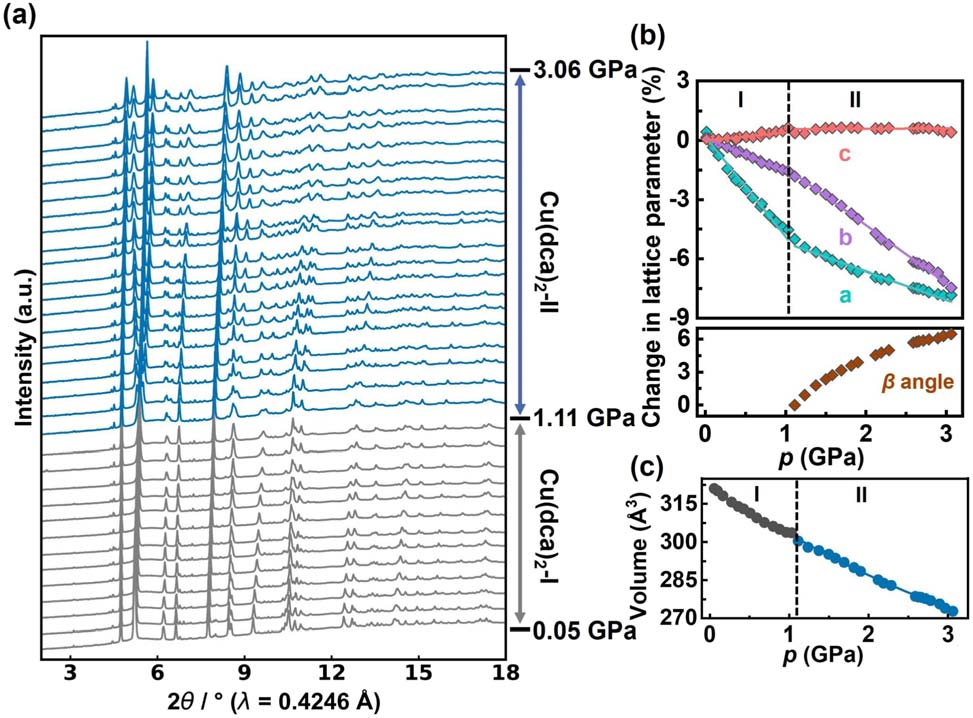
(a) HP-PXRD patterns of Cu(dca)_2_ in ascending order of pressure, illustrating the transition from orthorhombic to monoclinic phases. (b) Pressure-dependent evolution of Cu(dca)_2_ lattice parameters shown as percentage changes from ambient pressure values, determined by HP-PXRD. The lattice parameters *a*, *b* and *c* are fitted separately with linear functions for the orthorhombic phase I (Cu(dca)_2_-I) and monoclinic phase II (Cu(dca)_2_-II) regions, while the β angle of phase II is fitted with a third-order polynomial equation. Error bars are within the size of the data points. (c) Unit cell volume of Cu(dca)_2_ as a function of pressure. Grey points correspond to phase I and blue points to phase II. The data is fitted using the 2nd-order Birch–Murnaghan equation of state (BM EoS) using EoSFit7 GUI software.^44^

Cu(dca)_2_ has an orthorhombic structure under ambient conditions, constructed from CuN_6_ octahedra and trigonal dca^−^ linkers. A pronounced Jahn–Teller distortion occurs in the CuN_6_ octahedra due to the d^9^ electron configuration of the Cu^2+^ ions. This asymmetrical distortion is evidenced by significant bond length disparities; specifically, the axial Cu–N2 bond is elongated to 2.45 Å, while the equatorial Cu–N1 bond is considerably shorter at 1.92 Å. The axial Cu–N2 bond, oriented within the *a*–*b* plane of the crystal structure, indicates potentially weaker bonding in this direction. This structural feature suggests a theoretical basis for rapid compression along the *a*- and *b*-axes of the crystal lattice under pressure. Our HP-PXRD study of Cu(dca)_2_ confirms this hypothesis, as shown in Fig. 2.

Rietveld refinement of the HP-PXRD patterns shows that Cu(dca)_2_ preserves its initial orthorhombic phase, labelled Cu(dca)_2_-I, over a pressure range from 0.05 to 1.11 GPa. Subsequently, a structural phase transition occurs, leading to a monoclinic phase, Cu(dca)_2_-II, which persists up to pressures of 3.06 GPa. HP-PXRD patterns over these two phases are shown in Fig. 2(a). The splitting of peaks beyond 1.11 GPa is indicative of this symmetry-lowering process. The subtle volume discontinuity observed across the phase transition, as shown in Fig. 2(c), indicates that this is a first-order transition. This phase transition is reversible. Upon pressure reduction, the compound reverts from the Cu(dca)_2_-II phase back to its original orthorhombic configuration, Cu(dca)_2_-I (see SI Fig. S1).

The lattice parameters of Cu(dca)_2_ as a function of pressure are illustrated in Fig. 2(b). For phase I, the lattice parameter *c* shows a progressive increase with increasing pressure, while lattice parameters *a* and *b* show systematic decreases, clearly demonstrating that Cu(dca)_2_ undergoes pronounced anisotropic deformation under compression. The bulk modulus and uniaxial linear compressibility along each orthorhombic axis were determined for both phases of Cu(dca)_2_ using EoSFit7 and PASCal software, respectively.^45,46^ It shows that Cu(dca)_2_-I exhibits positive linear compressibility (PLC) along the *a*- and *b*-axes and negative linear compressibility (NLC) along the *c*-axis, with values of 42.3(8) TPa^−1^, 16.5(9) TPa^−1^ and −6.5(10) TPa^−1^, respectively, within the pressure range of 0.05 to 1.11 GPa. The calculated bulk modulus for Cu(dca)_2_-I is 14.9(4) GPa.

Cu(dca)_2_-II, while not exhibiting NLC behaviour, also exhibits a minimum PLC along the *c* axis with a value of 7.1(8) TPa^−1^ over the pressure range of 1.24 to 3.06 GPa; the corresponding PLC values along the *a*- and *b*-axes are 17.1(10) TPa^−1^ and 30.7(8) TPa^−1^, respectively. The bulk modulus of Cu(dca)_2_-II is 12.3(3) GPa, which is lower than that of Cu(dca)_2_-I, indicating reduced mechanical stiffness after the phase transition. While this behaviour is somewhat uncommon, it is not unprecedented and has been observed in other materials. For example, CrN exhibits an unexpected reduction of the bulk modulus of about 25% in the high-pressure orthorhombic phase compared to the low-pressure cubic phase at a transition pressure of ≈ 1 GPa.^47^ Similarly, Cu_2_O shows a phenomenon where the monoclinic high-pressure phase has a bulk modulus of 41(6) GPa, which is three times softer than the low-pressure cubic phase at 125(2) GPa.^48^ In the case of Cu(dca)_2_, this reduced stiffness can be attributed to the lower symmetry in phase II, which allows greater degrees of freedom within the framework and consequently enables more substantial deformation under pressure.^49^

**Fig. 3:**
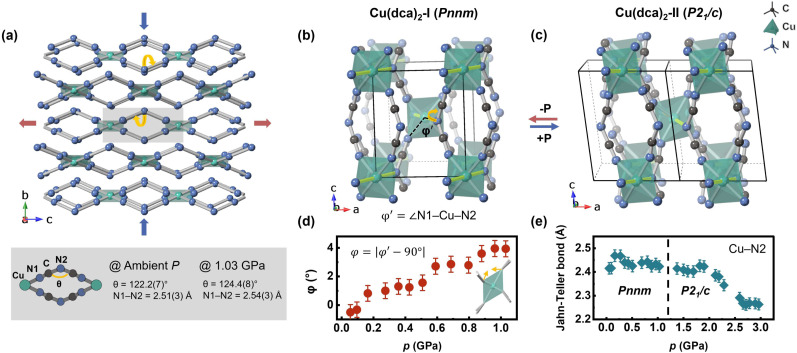
Structural deformation mechanisms in Cu(dca)_2_ under pressure. (a) The “wine-rack” topology of Cu(dca)_2_-I, with the axial Cu–N2 bonds omitted for clarity. This characteristic “wine-rack” motif governs the anisotropic deformation behaviour under compression, driving rapid contraction along the *a*- and *b*-axes while simultaneously inducing expansion along the perpendicular *c*-axis. The bottom panel shows the dca^−^ ligand geometry and coordination mode, with the internal angle *θ* increasing from 122.2(7)° at ambient pressure to 124.4(8)° at 1.03 GPa, illustrating the ligand deformation mechanism. (b) Crystal structure of Cu(dca)_2_-I at 0.05 GPa and (c) Cu(dca)_2_-II at 3.06 GPa, demonstrating the symmetry-breaking phase transition. The Jahn–Teller distorted Cu–N2 bonds are highlighted in yellow. During this transition, the terminal nitrogen atoms of the dca^−^ linkage become inequivalent, resulting in two distinct Cu–N1 bonds. (d) Evolution of the octahedral distortion parameter *φ* with increasing pressure, where *φ* quantifies the deviation of the N1–Cu–N2 angle from 90°. (e) Pressure-dependent behaviour of the long Jahn–Teller-distorted Cu–N2 bond length over the studied pressure range.

The key structural feature of Cu(dca)_2_-I that governs its anisotropic deformation under pressure is its “wine-rack” topology, illustrated in Fig. 3(a). This distinctive network architecture exhibits pronounced compressibility along the *a*–*b* plane, which results from the presence of comparatively weaker, elongated Cu–N2 bonds. Through an efficient flexible hinge mechanism, this compression transforms into expansion along the perpendicular *c*-axis direction.^50^ Detailed investigation of the structural deformation under pressure revealed an unexpected result: contrary to predictions, the Cu–N2 bond does not contract under pressure. As shown in Fig. 3(e), this bond maintains nearly constant length throughout the pressure range of 0.05–1.9 GPa. Instead, Cu(dca)_2_-I exhibits significant octahedral distortion that coincides with compression of the unit cell along the *a*- and *b*-axes, as quantified by the parameter *φ*. This parameter measures the deviation of the N1–Cu–N2 angle from the ideal 90°, and its evolution is shown in Fig. 3(d). To determine *φ*, the actual N1–Cu–N2 angle is calculated from the Rietveld refined structure and the difference from 90° is calculated according to eqn (1).

1 *φ* = |*φ*′ − 90°|

The observed octahedral distortion results from hinge-like tilting of the dca^−^ ligands, which act as the ‘soft’ component of the framework, deforming to accommodate pressure-induced stress throughout the unit cell. Accompanying this tilting deformation, changes in the dca^−^ ligand geometry are also evident. The dca^−^ ligand, which consists of a coniform linkage with two C≡N1 bonds, undergoes deformation through modulation of its angle. As rapid compression occurs along the *a*- and *b*-axes, the dca^−^ ligands flatten in these directions while simultaneously stretching along the perpendicular *c*-axis, thereby causing expansion of the unit cell in this direction. The angle *θ* increases from 122.2(7) to 124.4(8)° over the pressure range of 0.0 to 1.03 GPa. These two coordinated deformation mechanisms—octahedral distortion and ligand deformation—appear to be the primary mechanisms responsible for negative linear compressibility in Cu(dca)_2_-I.

At 1.1 GPa, Cu(dca)_2_ undergoes a structural transformation from an orthorhombic to a monoclinic phase. This phase transition introduces anisotropic character to the previously isotropic N1–C–N2–C–N1 bonds. Additionally, the angular parameter *φ*, which indicates octahedral distortion, no longer increases systematically with pressure due to the enhanced structural complexity of Cu(dca)_2_-II. In this higher-pressure phase, Jahn–Teller bond contraction emerges as the primary deformation mechanism, exhibiting a multi-stage behaviour. The Cu–N2 bond length shows distinct compression stages: initially remaining nearly constant from 0.05–1.9 GPa, followed by significant contraction of 0.16 Å between 1.9 and 2.7 GPa, and then leveling off at higher pressures, likely because other distortion mechanisms become dominant. However, due to the symmetry breaking and increased number of crystallographically distinct sites in the low-symmetry monoclinic structure, it is challenging to establish clear correlations between different deformation mechanisms and identify distinct pressure regimes. These observations suggest that Cu(dca)_2_-II, which possesses a denser structure than Cu(dca)_2_-I, accommodates compression primarily through contraction of Jahn–Teller bonds in the pressure range of 1.9–2.7 GPa. This deformation mechanism differs from that observed in Cu(dca)_2_-I, where deformation occurs through tilting of the binding ligands within the more spacious intra-framework voids. Despite its denser packing, Cu(dca)_2_-II exhibits a lower bulk modulus than Cu(dca)_2_-I, indicating a more compliant structure. This softening can be attributed to the loss of the directional rigidity that characterizes phase I along the *c*-axis, combined with the reduced symmetry in phase II, which allows greater degrees of freedom within the framework and consequently enables more substantial deformation under pressure.

Cu(tcm)_2_, like Cu(dca)_2_, has a rutile-like coordination framework. This compound consists of octahedral Cu^2+^ cations and trigonal tcm^−^ anions. Under ambient conditions, Cu(tcm)_2_ shows an orthorhombic structure with space group *Pmna*. A characteristic Jahn–Teller distortion is observed, with the Jahn–Teller axis lying within the *b*–*c* plane, leading to an elongation of the axial Cu–N2 bonds by 0.47 Å with respect to their equatorial Cu–N1 counterparts. Despite these similarities, Cu(tcm)_2_ differs from Cu(dca)_2_ in terms of its structural arrangement. It has a doubly interpenetrating structure, as opposed to the single network seen in Cu(dca)_2_. This interpenetration arises from the increased distance between the octahedral nodes in Cu(tcm)_2_. Within Cu(tcm)_2_, Cu^2+^ cations are separated by extended paths of 5 atoms (N–C1–C2–C1–N). Conversely, Cu(dca)_2_ features two distinct pathways between the Cu centers, namely N1–C–N2–C–N1 and N1–C–N2 with chains of 5 and 3 atoms, respectively. While the 5-atom pathway matches the length of the connections in Cu(tcm)_2_, the shorter 3-atom connection in Cu(dca)_2_ significantly reduces the void space, thereby restricting the potential for interpenetration.^37^

**Fig. 4:**
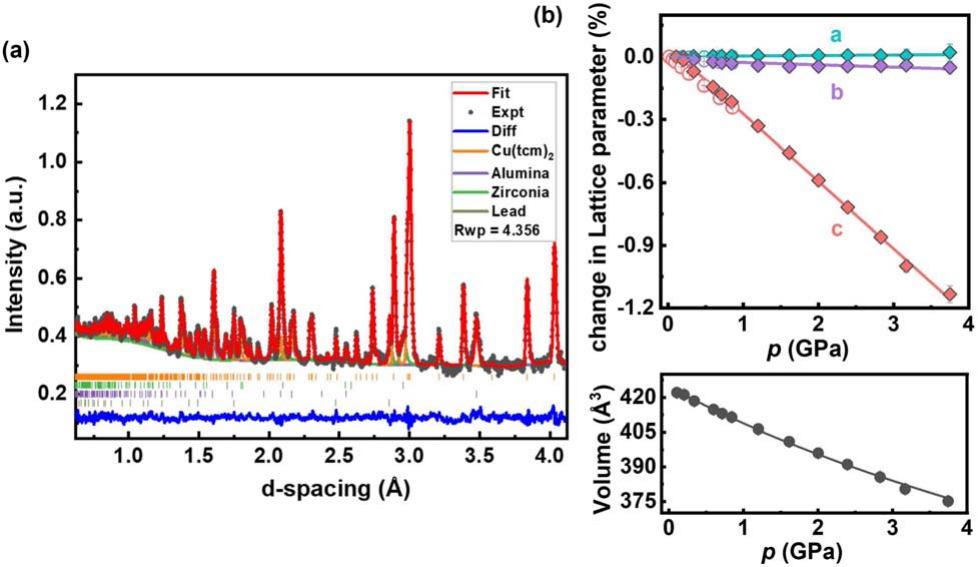
(a) Neutron diffraction pattern of Cu(tcm)_2_ at 0.11 GPa, fitted using Rietveld refinement. The experimental data are displayed in black, the fit in red, and the residuals in blue. The calculated signals for alumina, zirconia, and lead, which come from anvil and pressure marker, are represented by purple, green, and dark green lines, respectively, while the permitted reflections for each phase are represented by vertical bars. The excellent visual agreement demonstrates the quality of the refinement. (b) Variation of the lattice parameters of Cu(tcm)_2_ in response to increasing pressure. The HP-PNRD and HP-PXRD data points are represented by solid and open diamonds, respectively. The lattice parameters are linearly fitted while the volume data is fitted using the 2nd-order Birch–Murnaghan equation of state (BM EoS) using EoSFit7 GUI software.^44^

Rietveld refinement of HP-PNRD patterns reveals that Cu(tcm)_2_ maintains its orthorhombic structure at ambient conditions and over a wide pressure range of 0–3.75 GPa (see Fig. 4 and S4 in SI). The bulk modulus of Cu(tcm)_2_ within this pressure range is 24.5(4) GPa, significantly higher than that of Cu(dca)_2_-I, which is 14.9(4) GPa over 0.05–1.11 GPa. This higher modulus indicates greater stiffness of the former, enabling it to retain its initial structure under extreme conditions, but limiting its structural response to applied pressure. Regarding lattice deformation under pressure, Cu(tcm)_2_ exhibits positive linear compressibility (PLC) along the *c*-axis of 29.6(4) TPa^−1^ and along the *b*-axis of 1.1(3) TPa^−1^. It also displays zero linear compressibility (ZLC) along the *a*-axis with a value of −0.1(2) TPa^−1^. Unlike Cu(dca)_2_, the compound does not show substantial negative linear compressibility. Instead, it exhibits stable ZLC behaviour along the *a*-axis and very small PLC of 1.1(3) TPa^−1^ along the *b*-axis over a wide pressure range. This combination results in a near-zero area compression in the *a*–*b* plane of 1.0(5) TPa^−1^, a property rarely observed in materials.^51^ Such exceptional mechanical behaviour, with compression along the *c*-axis while maintaining near-zero area compression in the *a*–*b* plane, makes Cu(tcm)_2_ promising for potential applications in ultrasensitive pressure sensors and actuators where directional mechanical response is desirable.^52^

Although octahedral and dca^−^ ligand distortions are major pressure-induced deformations influencing the NLC behaviour in Cu(dca)_2_, these phenomena are not detected in Cu(tcm)_2_. This discrepancy is due to the combined effects of spatial constraints within the framework and the inherent rigidity of the ligands in the latter compound. On the one hand, the doubly interpenetrating network structure of Cu(tcm)_2_ increases the density, thereby preventing any potential ligand tilt-induced octahedral distortion. On the other hand, the coordination geometry of tcm^−^ ligands limits the extent of flexibility found in Cu(dca)_2_. In contrast to dca^−^, tcm^−^ possesses a planar, trigonal geometry that is notably rigid. Its interaction with metal centers occurs strictly through its terminal nitrogen donors. This stable trigonal geometry enables tcm^−^ to maintain its structural integrity under compression, resisting distortion of its internal geometry.

**Fig. 5:**
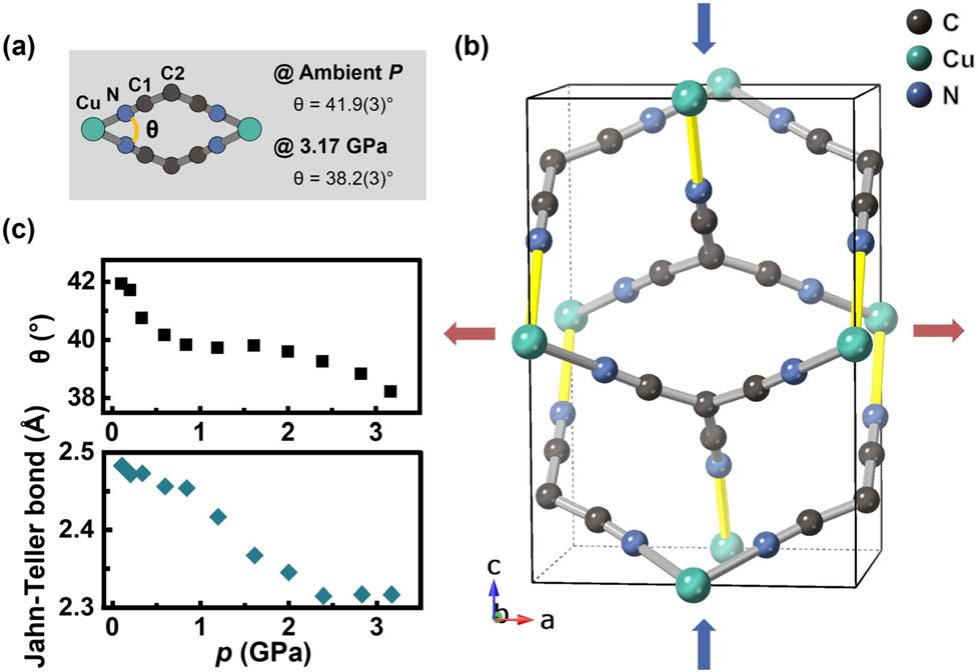
Pressure-induced structural deformation mechanisms in Cu(tcm)_2_. (a) Local coordination environment showing the Cu centre and tcm^−^ ligand with the inter-network torsion angle *θ* at ambient pressure and 3.17 GPa. (b) Crystal structure viewed along the *a*-axis, showing the doubly interpenetrating rutile-like networks. The Jahn–Teller distorted Cu–N bonds are highlighted in yellow. (c) Pressure dependence of the inter-network torsion angle *θ* and Jahn–Teller bond length, demonstrating the multi-stage deformation behaviour under compression.

The pronounced anisotropic deformation in Cu(tcm)_2_ is driven by compaction of interpenetrating networks and Jahn–Teller bonds. As shown in Fig. 5, the interpenetrating structure has an architectural feature with substantial intraframework space along the *c*-axis, which facilitates rapid contraction of the unit cell along this axis under pressure. The extent of deformation within the Cu(tcm)_2_ crystal structure is characterized by the variation in the torsion angle *θ*, which measures the twist between two opposing planes of planar tcm^−^ ions within the two interpenetrating networks. The value of *θ* is influenced by both the distance between the two interpenetrating networks and changes in the *a* lattice parameter. Throughout the pressure range, the deformation exhibits a multi-stage mechanism in *θ*, correlating with pressure increase and suggesting a closer approach of the two interpenetrating networks. Analysis reveals three distinct compression stages: (i) 0–1 GPa, where compression occurs primarily *via* the angle *θ*; (ii) 1–2.5 GPa, where deformation is dominated by Jahn–Teller bond contraction; and (iii) above 2.5 GPa, where the torsion angle mechanism becomes dominant again. Furthermore, the Jahn–Teller-distorted long bond in Cu(tcm)_2_, located in the *b*–*c* plane, contributes to the overall compressibility. Characterized by its length and relative weakness, this bond consistently contracts upon compression, a behaviour evident in Fig. 5, and shows a leveling off at approximately 2.5 GPa. This multi-stage compression behaviour is similar to that observed in Cu(dca)_2_-II, where different deformation mechanisms dominate at different pressure ranges. The *c*-axis of Cu(tcm)_2_ is connected to the *a*-axis *via* a ‘wine-rack’ packing of atoms. This configuration enables the efficient transformation of rapid compression along the *c*-axis into expansion in the perpendicular directions. As a result, ZLC is observed along the *a*-axis. Similarly, the negligible positive linear compressibility (PLC) magnitude along the *b*-axis can be interpreted as a consequence of the network's compression-induced reconfiguration along the *c*-axis.

## 4 Conclusion

In conclusion, Cu(dca)_2_ and Cu(tcm)_2_, as structural analogues of rutile, both display anisotropic compression behaviour, though to varying degrees. Specifically, Cu(dca)_2_ demonstrates a pronounced negative linear compressibility (NLC) of −6.5(10) TPa^−1^ along the *c*-axis across a pressure range of 0.05 to 1.11 GPa. By contrast, Cu(tcm)_2_ shows zero linear compressibility (ZLC) with a magnitude of −0.1(2) TPa^−1^ along the *a*-axis, maintained over a wider pressure range from 0 to 3.75 GPa. The appearance of NLC/ZLC behaviour in these compounds can be attributed to their ‘wine-rack’ topology, which effectively translates rapid compression in one direction into expansion in the perpendicular direction. The differences in compressibility magnitudes between these two compounds can be explained by considering the disparities in their connecting ligands and structural configurations. Cu(dca)_2_ features a single rutile-like structure formed by flexible dca^−^ ligands. This structure, compared to the interpenetrating architecture of Cu(tcm)_2_ linked by rigid tcm^−^ ligands, undergoes more pronounced deformation upon compression. This results in a greater magnitude of NLC but also a more rapid structural collapse. Furthermore, Cu(dca)_2_ undergoes a first-order phase transition from an orthorhombic to a monoclinic structure above 1.11 GPa. Conversely, Cu(tcm)_2_ retains its initial orthorhombic structure over the entire pressure range without undergoing any phase transition.

Both Cu(dca)_2_ and Cu(tcm)_2_ exhibit a notable elongation of the Cu–N2 bonds, attributed to the Jahn–Teller effect. However, their responses to compression vary distinctly. Specifically, Cu(tcm)_2_ undergoes a considerable Cu–N2 bond contraction when compressed, a feature not observed in Cu(dca)_2_. This discrepancy arises from the flexibility of the dca^−^ ligands present in Cu(dca)_2_, which deform to accommodate compressive stress, preventing the bonds from contracting. Although Cu(dca)_2_ does not demonstrate direct Jahn–Teller bond contraction, the effect nevertheless plays a critical role in this compound. The Jahn–Teller distortion creates anisotropic coordination environments that generate considerable intraframework space along the *a*- and *b*-axes, facilitating the deformation of the dca^−^ ligands. This process enhances the compressibility along these axes, further emphasising the NLC behaviour of Cu(dca)_2_ under pressure.

The predominant pressure-induced deformations in Cu(dca)_2_ and Cu(tcm)_2_ differ significantly. For Cu(dca)_2_-I, the main changes are octahedral and dca^−^ ligand distortions, whereas Cu(tcm)_2_ primarily undergoes compaction of interpenetrating networks and Jahn–Teller bond contraction. This variance underlines the distinct structural responses of these copper compounds under pressure, demonstrating the diversity of their physical properties despite their shared rutile-like topology.

These findings establish important design principles for engineering NLC materials. The interplay between three key factors emerges as crucial: (i) framework topology (wine-rack motif with single *vs.* interpenetrating networks), (ii) ligand flexibility (flexible dca^−^*vs.* rigid tcm^−^), and (iii) electronic effects (Jahn–Teller distortion). The Cu(dca)_2_ system demonstrates that combining flexible ligands with Jahn–Teller-active metal centers in a single wine-rack network topology can achieve NLC values (−6.5 TPa^−1^) that are significantly higher than conventional inorganic rutile materials (typically <2 TPa^−1^), while Cu(tcm)_2_ shows ZLC and near-zero area compressibility phenomenon with exceptional stability over wide pressure ranges. These insights provide a rational framework for designing next-generation mechanical metamaterials with tailored compressibility responses, potentially advancing applications in pressure-resistant optical devices, ultrasensitive sensors, and mechanical actuators where precise directional responses are essential.

## Author contributions

M. C.: conceptualization, methodology, investigation, formal analysis, writing – original draft. H. L. B. B.: investigation, writing – review & editing. D. D.: investigation, writing – review & editing. N. P. F.: investigation, writing – review & editing. C. J. R.: investigation, writing – review & editing. A. B. C.: conceptualisation, investigation, supervision, project administration, funding acquisition, writing – review & editing.

## Conflicts of interest

There are no conflicts to declare.

## Supplementary Material

SC-OLF-D5SC04854K-s001

## Data Availability

Supplementary information (SI): crystallographic data, high-pressure diffraction patterns, compressibility calculations, elemental analysis, and SEM characterization. See DOI: https://doi.org/10.1039/d5sc04854k.
