# Supplementary material for: Negative and zero linear compressibility in copper dicyanamide and tricyanomethanide
Source: Chem Sci. 2025 Dec 17;17(7):3700–7. doi: 10.1039/d5sc04854k (PMC12757784; doi:10.1039/d5sc04854k)
Supplement: SC-017-D5SC04854K-s001 [file SC-017-D5SC04854K-s001.pdf]

Supplementary Information (SI) for Chemical Science.
This journal is © The Royal Society of Chemistry 2025

SUPPORTING INFORMATION

Negative and Zero Linear Compressibility in Copper Dicyanamide and Tricyanomethanide

Muzi Chen,*[abc] Hanna L. B. Boström,[de] Dominik Daisenberger,[f] Nicholas P. Funnell,[g] Christopher J. Ridley,[gh] and Andrew B. Cairns*[ab]

[a] Department of Materials, Imperial College London, Royal School of Mines, Exhibition Road, SW7 2AZ, London, U.K

[b] London Centre for Nanotechnology, Imperial College London, London SW7 2AZ, UK

[c] Department of Heterogeneous Catalysis, Max-Planck-Institut für Kohlenforschung, Kaiser-Wilhelm-Platz 1, 45470 Mülheim an der Ruhr, Germany

[d] Department of Chemistry, Stockholm University, Svante Arrhenius väg 16C, SE-106 91, Stockholm, Sweden

[e] Wallenberg Initiative Materials Science for Sustainability, Department of Chemistry, Stockholm University, SE-114 18, Stockholm, Sweden

[f] Diamond Light Source Ltd., Harwell Campus, Didcot OX11 0DE, U.K

[g] ISIS Neutron and Muon Source, Rutherford Appleton Laboratory, Harwell Campus, Didcot OX11 0QX, U.K

[h] Neutron Scattering Division, Oak Ridge National Laboratory, Oak Ridge, TN 37831, USA.

∗To whom correspondence should be addressed;

E-mail: muzichen@kofo.mpg.de

E-mail: a.cairns@imperial.ac.uk

Contents

1. Bulk modulus and linear compressibility 3

2. Crystallographic details 7

3. Elemental analysis 10

4. Variable-pressure X-ray diffraction data of $Cu(dca)_2$ 12

5. Variable-pressure neutron diffraction data of $Cu(tcm)_2$ 16

6. Variable-pressure X-ray diffraction data of $Cu(tcm)_2$ 19

7. References 22

1. Bulk modulus and linear compressibility

Birch-Murnaghan Fitting: Variable-pressure unit cell volume data for all phases over the entire stability range were fitted to the second-order Birch-Murnaghan equation of state (EoS) using EoSFit7-GUI. [1] The applicability of the second-order fit was determined by inspection of the f-F plots, which visualize the normalized pressure *versus* Eulerian strain data for each phase. A horizontal trend was observed within experimental error for all phases in these plots, indicating that a second-order Birch-Murnaghan fit sufficiently accounted for the pressure-volume data.[2] Optimal fits to the pressure-volume data (Figure 1) enabled calculation of the bulk modulus (B_{Pi}, where Pi is the initial pressure of the measurement) and its pressure derivative (B′) for each phase with reasonable uncertainty, as shown in Table S1.

Powder Diffraction Profile Fitting: Powder diffraction patterns of $Cu(dca)_2$ and $Cu(tcm)_2$ were analyzed by Rietveld refinement using the TOPAS software.[3,4] Initial structural parameters were derived from previously reported single-crystal X-ray data retrieved from the CCDC database for these complexes at ambient conditions.[5,6] These parameters, including lattice constants, atomic coordinates, and B_{iso} values, were subsequently refined based on our collected diffraction patterns and varied with applied pressure. For $Cu(dca)_2$ refinement, geometric constraints were initially applied, including constraining the N1-C1-N2 angle to 180° to maintain the linear geometry of the nitrile groups and constraining C-N bond lengths. For $Cu(tcm)_2$ structure refinement, the tcm^- ligands ($C_4N_3^-$) were initially constrained as rigid planar bodies to preserve their trigonal planar structure. However, all geometric constraints were subsequently removed for both compounds as they did not significantly affect the refinement results, and the final structural models were refined without geometric restraints. The high-pressure (HP) monoclinic phase structure of $Cu(dca)_2$ was derived by modifying the crystal structure of HP-$Co(dca)_2$ reported previously.[7] Given that $Cu(dca)_2$ and $Co(dca)_2$ are isostructural with identical rutile-like structures under ambient conditions and undergo phase transitions at the same pressure (1.11 GPa), we hypothesized they would share similar structures in the high-pressure regime. Satisfactory fitting results confirmed this hypothesis, validating our approach to obtaining the HP-$Cu(dca)_2$ structure.

Lattice parameters and compressibility: Lattice parameters were determined through Rietveld refinement of the diffraction patterns. The coefficient of linear compressibility for each phase was calculated using PASCal by correlating these lattice parameters with applied pressure.[8] Results presented in Table S1 demonstrate that $Cu(dca)_2$ exhibits two distinct sets of linear compressibilities corresponding to its orthorhombic and monoclinic phases. Unlike $Cu(dca)_2$, $Cu(tcm)_2$ maintains its structure without phase transitions throughout the measured pressure range. It should be noted that data were collected from two different experimental techniques—neutron and X-ray diffraction—and the calculated linear compressibility values are influenced by the pressure steps and ranges employed. A notable discrepancy exists between the results: the neutron diffraction experiment yielded consistently higher B_{Pi} values compared to X-ray diffraction, suggesting a more rigid structure of $Cu(tcm)_2$ in the high-pressure regime.

Table S1: Compressibility and bulk modulus of $Cu(dca)_2$ and $Cu(tcm)_2$ calculated within different pressure ranges.

Materials	K_a (Tpa^{-1})	K_b (Tpa^{-1})	K_c (Tpa^{-1})	B_{pi} (GPa)	B′	Range (GPa)	Data points
LP-$Cu(dca)_2$	42.3(7)	16.4(4)	-6.45(0)	14.9(4)	4	0.05-1.11	16
HP-$Cu(dca)_2$	17.1(10)	30.7(8)	7.1(8)	12.3(3)	4	1.24-3.06	20
$Cu(tcm)_2$*	−0.1(2)	1.1(3)	29.6(4)	24.5(4)	4	0.11-3.75	13
$Cu(tcm)_2$	−0.89(6)	7.04(13)	26.4(3)	27.7(4)	4	0.02-0.86	8

*Obtained from neutron diffraction.

Experimental Timeline: $Cu(tcm)_2$ was initially measured at ESRF as part of a broader study to 0.86 GPa. Subsequently, neutron beamtime was obtained at ISIS to acquire structural data with higher sensitivity to light elements and extend the pressure range to 3.75 GPa. Later, beamtime was obtained at Diamond Light Source for the $M(dca)_2$ series investigation, which unfortunately did not include additional time to remeasure $Cu(tcm)_2$. The previously collected ESRF and ISIS data for $Cu(tcm)_2$ were therefore combined with the Diamond Light Source data for $Cu(dca)_2$ for the comparative study presented here.

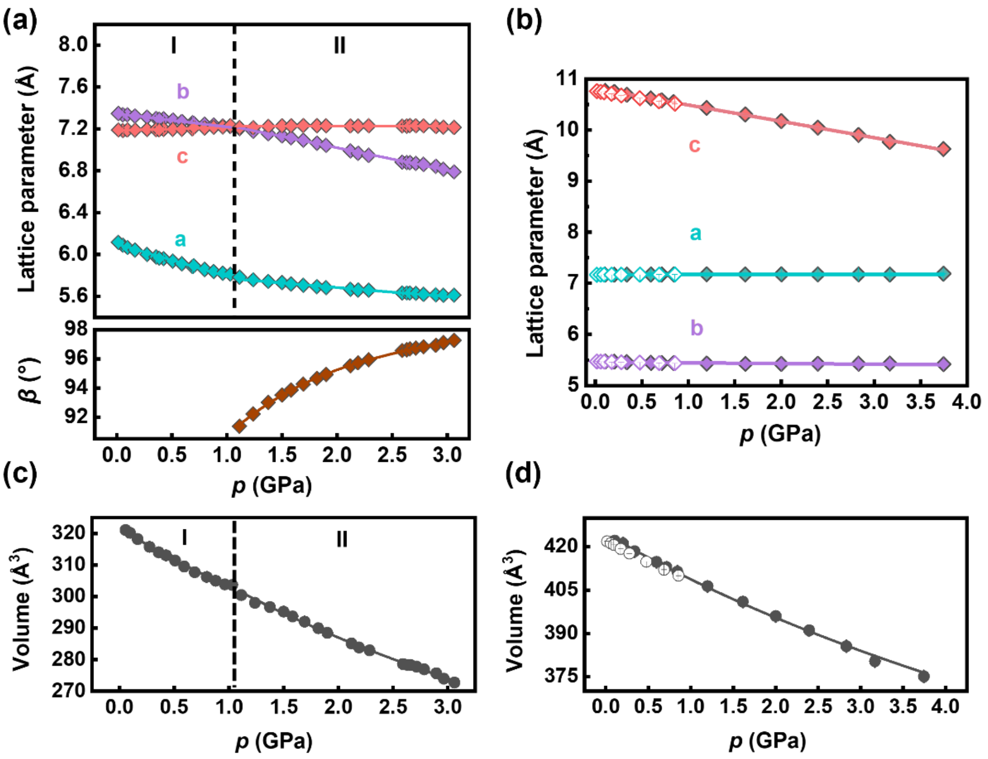

Figure S1: Pressure-dependent lattice parameters and volumes for $Cu(dca)_2$ and $Cu(tcm)_2$. (a) Lattice parameters of $Cu(dca)_2$ determined by HP-PXRD, with linear fits for orthorhombic phase I and monoclinic phase II regions, and third-order polynomial fit for the β angle of phase II. (b) Lattice parameters of $Cu(tcm)_2$ from HP-PNRD (solid diamonds) and HP-PXRD (open diamonds) with linear fits. (c) & (d) Unit cell volumes fitted using the second-order Birch-Murnaghan equation of state for $Cu(dca)_2$ and $Cu(tcm)_2$, respectively.

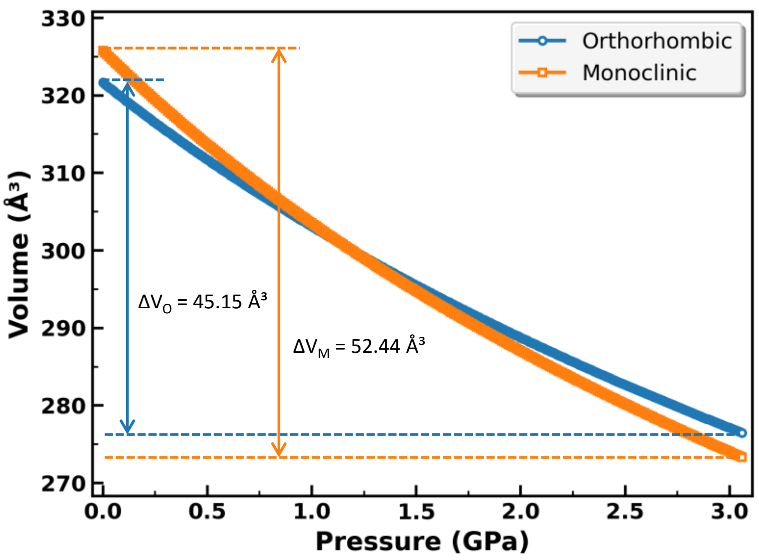

Figure S2: Birch-Murnaghan equation of state fits for $Cu(dca)_2$ orthorhombic (blue) and monoclinic (orange) phases extrapolated over 0-3.06 GPa. Dashed lines show volumes at 3.06 GPa, with ΔVO = 45.15 Å³ and ΔVM = 52.44 Å³, demonstrating lower density in the monoclinic phase.

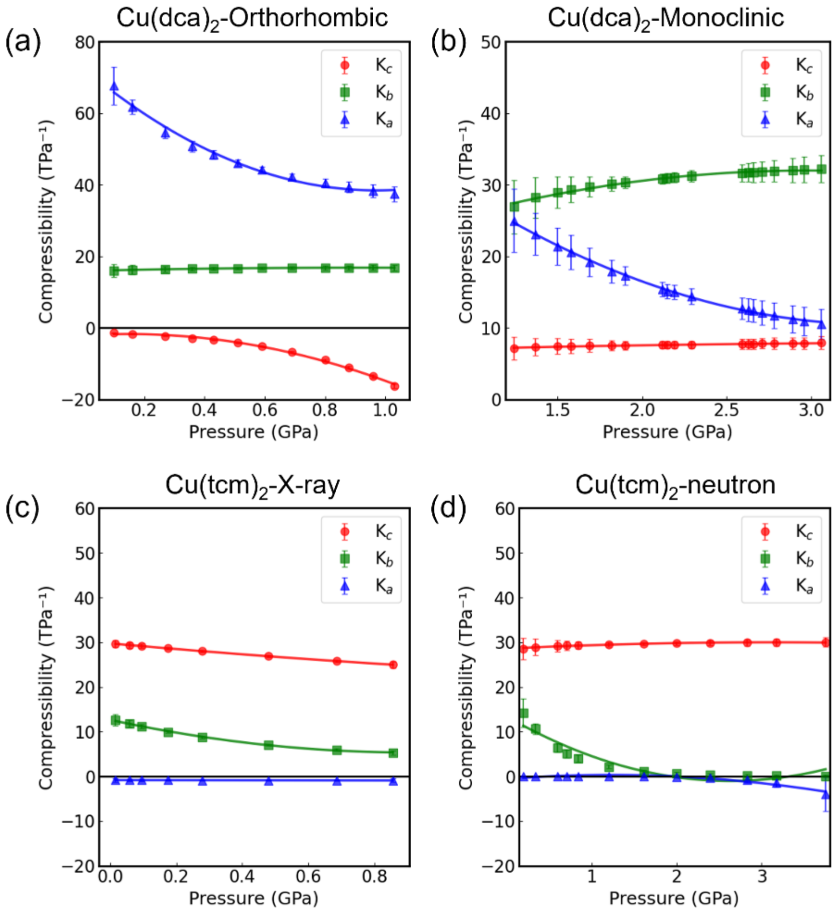

Figure S3: Compressibility as a function of pressure for $Cu(dca)_2$ and $Cu(tcm)_2$. (a) $Cu(dca)_2$ in its orthorhombic phase and (b) monoclinic phase. (c) $Cu(tcm)_2$ measured by X-ray diffraction and (d) neutron diffraction. All data were fitted using 2nd order polynomial functions.

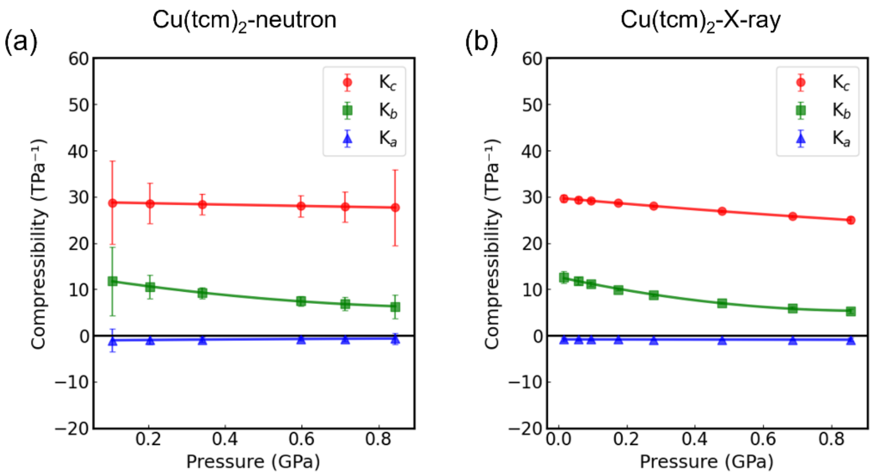

Figure S4: Comparison of compressibility values for $Cu(tcm)_2$ as a function of pressure obtained from (a) neutron diffraction and (b) X-ray diffraction measurements within overlapping pressure ranges. The compressibility along each crystallographic axis (K_a, K_b, K_c) shows good agreement between the two techniques within experimental uncertainties. All compressibility values were fitted using 2nd order polynomial fitting. The larger error bars in the neutron data reflect the smaller number of data points available for fitting in the low-pressure region.

2. Crystallographic details

Table S2: Crystallographic details determined by Rietveld refinement of X-ray diffraction data for $Cu(dca)_2$ at 0.05 GPa.

Space group	*Pnnm*				
a / Å	6.0900(7)				
b / Å	7.3367(8)				
c / Å	7.1855(12)				
β/ °	90				
V / Å^3	321.05(7)				
R_{wp}	1.59%				
**Atom**	**occ**	***x***	***y***	***z***	**B_{iso}* / Å^2**
C	1	0.245(5)	0.360(2)	0.158(3)	1.7(2)
Cu	1	0	0	0	1.7(2)
N1	1	0.305(4)	0.421(2)	0.304(3)	1.7(2)
N2	1	0.177(6)	0.294(5)	0	1.7(2)

* B_{iso} constrained to be equal for all atoms.

Table S3: Crystallographic details determined by Rietveld refinement of X-ray diffraction data for $Cu(dca)_2$ at 1.37 GPa.

Space group	$P2_1/c$				
a / Å	5.7453(8)				
b / Å	7.1564(8)				
c / Å	7.226(2)				
β/ °	93.001(10)				
V / Å^3	296.68(8)				
R_{wp}	1.59%				
**Atom**	**occ**	***x***	***y***	***z***	**B_{iso}* / Å^2**
C1	1	0.234(15)	0.138(7)	0.324(13)	1.0(4)
C2	1	−0.235(14)	−0.120(8)	0.349(13)	1.0(4)
Cu	1	0	0	0	1.0(4)
N1	1	0.317(12)	0.096(6)	0.183(9)	1.0(4)
N2	1	0.144(11)	0.184(7)	0.479(15)	1.0(4)
N3	1	−0.308(10)	−0.074(7)	0.208(8)	1.0(4)

* B_{iso} constrained to be equal for all atoms.

Table S4: Crystallographic details determined by Rietveld refinement of neutron diffraction data for $Cu(tcm)_2$ at 0.02 GPa.

Space group	*Pmna*
a / Å	7.16771(12)
b / Å	5.46999(11)
c / Å	10.7605(2)
β/ °	90
V / $Å^3$	421.892(14)
R_{wp}	0.80%

Atom	occ	x	y	z	B_{iso}* / $Å^2$
N1	1	0.2096(4)	0.2076(9)	0.4318(5)	1.17(6)
N2	1	0.5	0.7539(12)	0.2005(5)	1.17(6)
C1	1	0.3373(6)	0.2925(11)	0.3914(5)	1.17(6)
C2	1	0.5	0.5971(15)	0.2756(9)	1.17(6)
C3	1	0.5	0.399(2)	0.3543(10)	1.17(6)
Cu	1	0	0	0.5	1.17(6)

* B_{iso} constrained to be equal for all atoms.

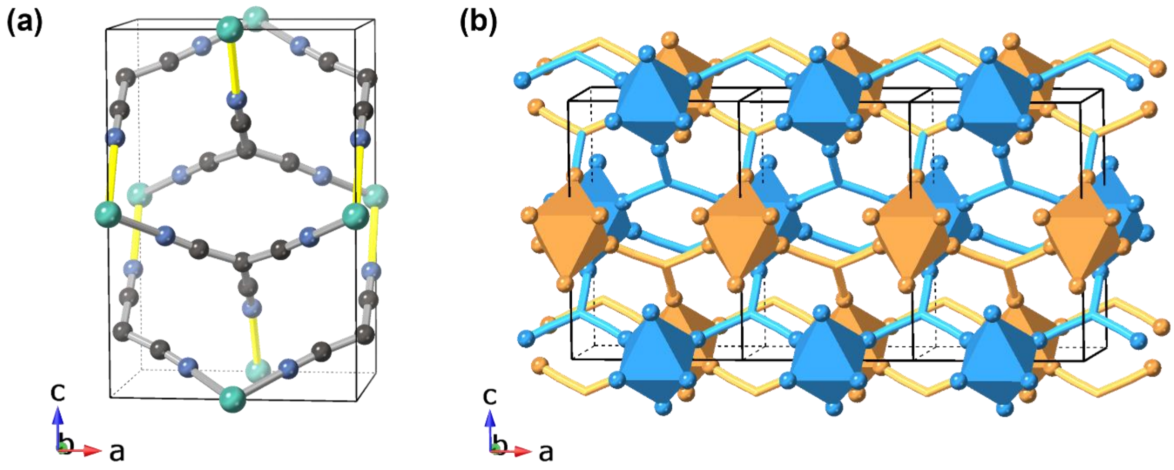

Figure S8: Crystal structure of $Cu(tcm)_2$ viewed along the *a*-axis, showing (a) the ball-and-stick representation (reproduced from Fig 5(b) for comparison) and (b) the octahedral representation of the doubly interpenetrating rutile-like networks. The blue and orange octahedra represent the two-interpenetrating network.

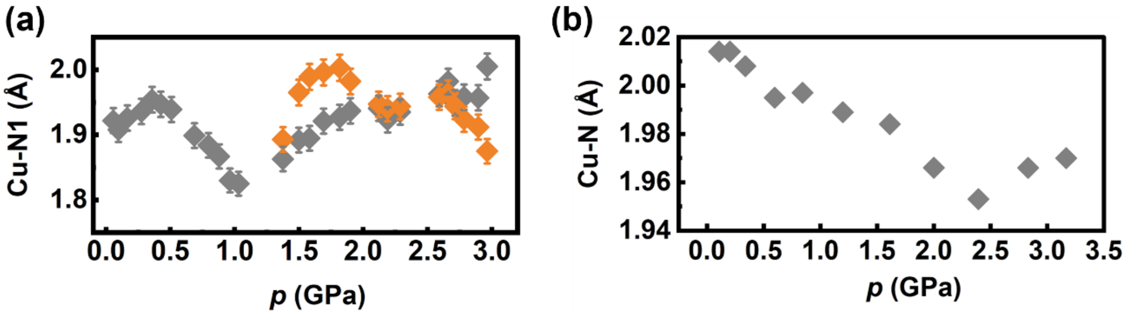

Figure S9: Pressure dependence of the Cu-N1 bond lengths (non Jahn-Teller bonds) for (a) $Cu(dca)_2$ and (b) $Cu(tcm)_2$. Gray diamonds represent the orthorhombic phase, while orange diamonds in (a) represent the monoclinic phase of $Cu(dca)_2$.

3. Elemental analysis

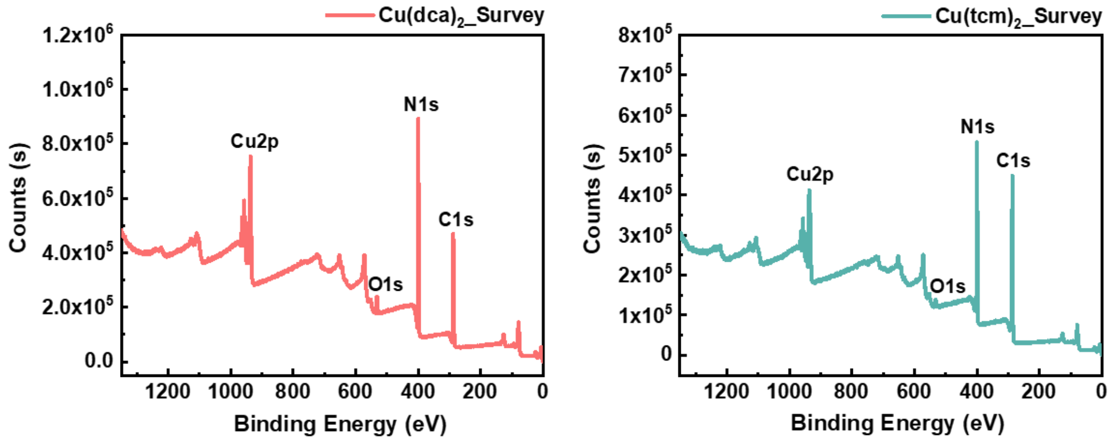

Name/atomic %	M	C	N	O	Total
Cu(tcm)$_2$	5.99	55.46	37.09	1.45	99.99
Cu(dca)$_2$	8.43	37.51	49.20	2.48	97.62

Figure S5: XPS survey spectra of Cu(dca)$_2$ and Cu(tcm)$_2$ confirming the elemental composition of both samples. The spectra show the presence of Cu, C, N, and minimal O content, indicating high sample purity with negligible impurities. The measurements were performed using a K-Alpha XPS system (Thermo Scientific) with a spot size of 400 μm and pass energy of 200.0 eV.

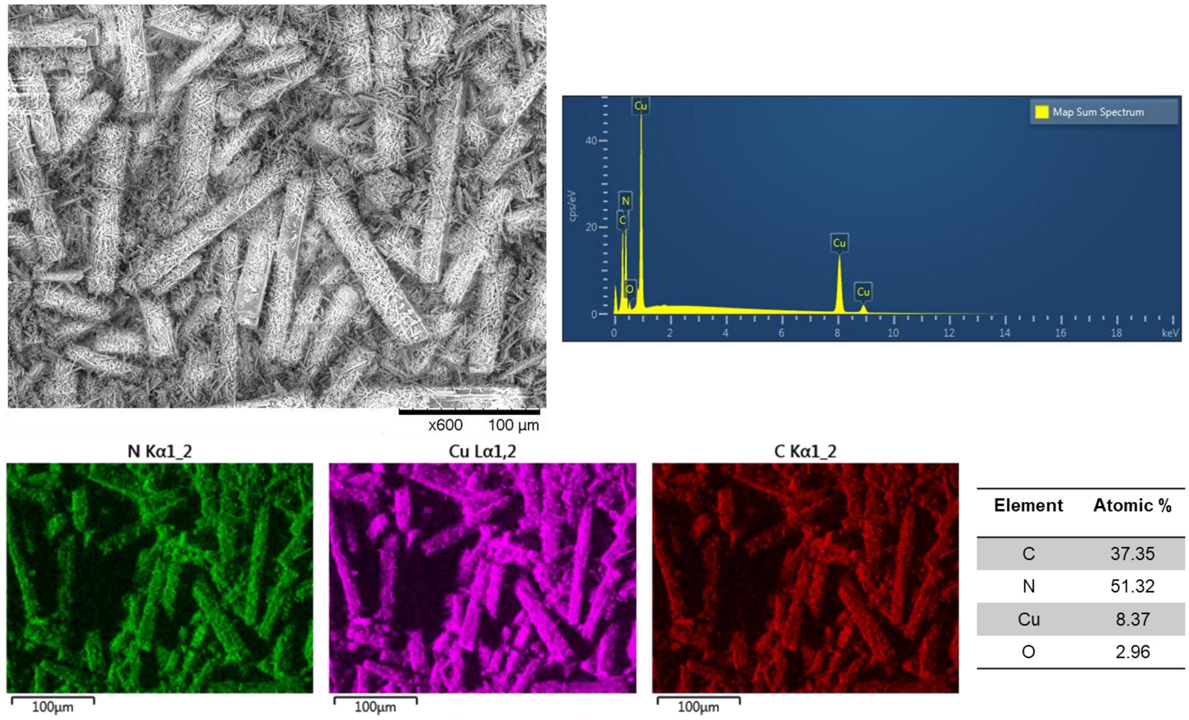

Element	Atomic %
C	37.35
N	51.32
Cu	8.37
O	2.96

Figure S6. SEM-EDX characterization of Cu(dca)$_2$. Top left: SEM image showing the morphology of Cu(dca)$_2$ microcrystals. Top right: EDX spectrum showing the presence of Cu, C, N, and O. Bottom: Elemental mapping images showing the spatial distribution of N (green), Cu (magenta), and C (red) across the sample. The table shows the atomic percentages of each element.

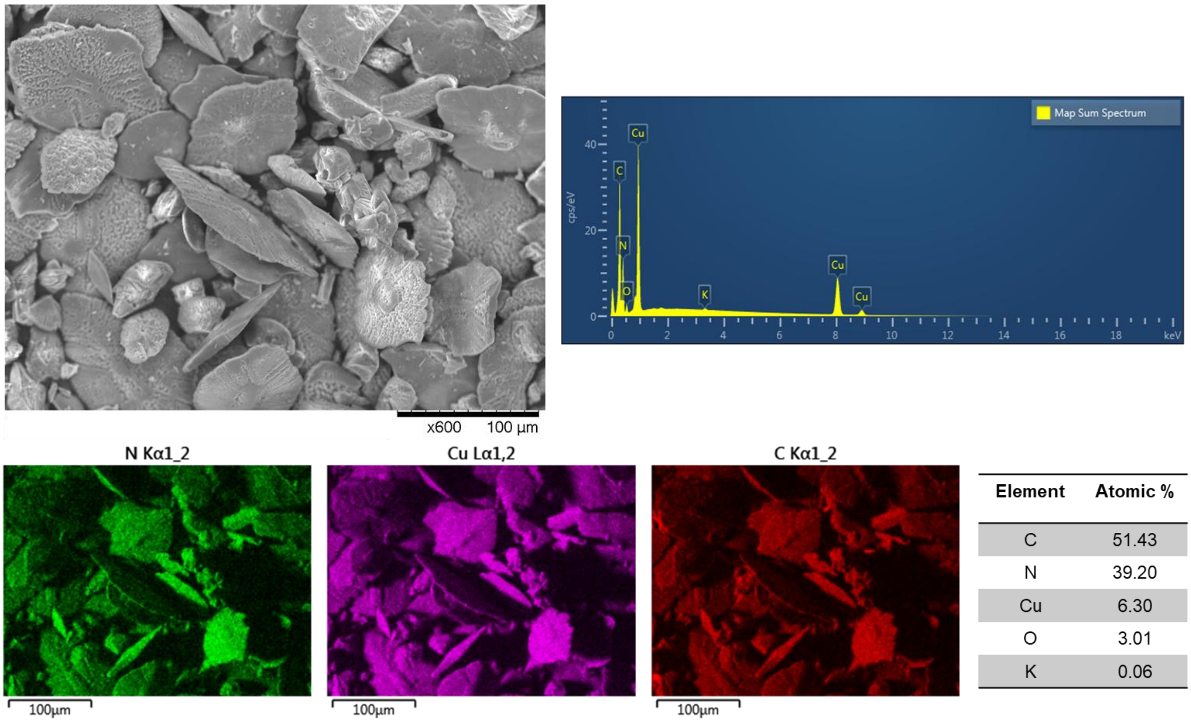

Element	Atomic %
C	51.43
N	39.20
Cu	6.30
O	3.01
K	0.06

Figure S7. SEM-EDX characterization of $Cu(tcm)_2$. Top left: SEM image showing the morphology of $Cu(dca)_2$ microcrystals. Top right: EDX spectrum showing the presence of Cu, C, N, and O. Bottom: Elemental mapping images showing the spatial distribution of N (green), Cu (magenta), and C (red) across the sample. The table shows the atomic percentages of each element.

Table S5: Elemental analysis results for $Cu(dca)_2$ and $Cu(tcm)_2$

Compound	Formula	Element	Mass-%	Atomic-%	Atomic ratio
$Cu(dca)_2$	CuC_4N_6	C	24.15	36.6	3.98
		N	41.8	54.2	5.91
		Cu	32.1	9.2	1
$Cu(tcm)_2$	CuC_8N_6	C	39.01	53.7	8.03
		N	33.5	39.6	5.92
		Cu	25.7	6.7	1

*Elemental analysis was performed by Mikroanalytisches Labor Pascher (Remagen, Germany). For C and N, combustion methods were used. For Cu, digestion and detection by ICP-AES were performed.

4. Variable-pressure X-ray diffraction data of $Cu(dca)_2$

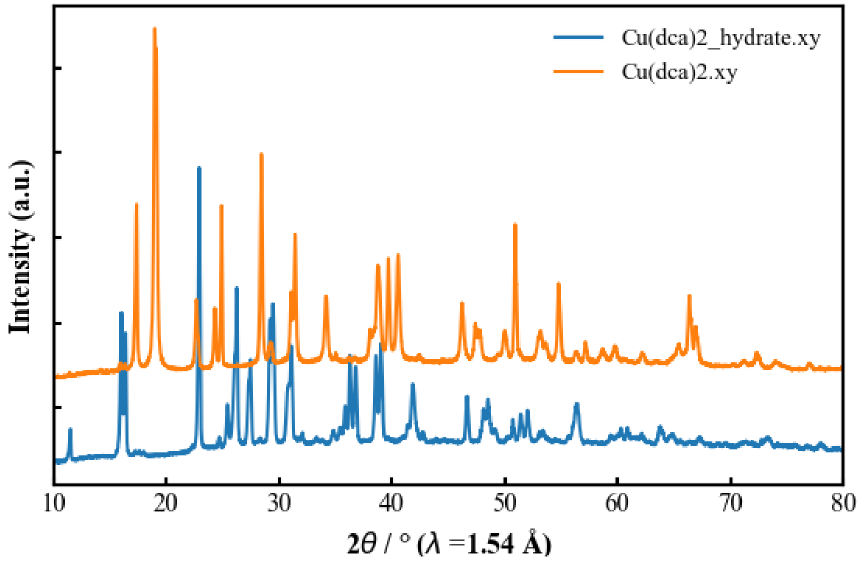

Figure S10: PXRD patterns comparison of hydrated $Cu(dca)_2$ as synthesized (blue) and anhydrous $Cu(dca)_2$ after vacuum treatment (orange), demonstrating the phase transition from the hydrated to anhydrous form.

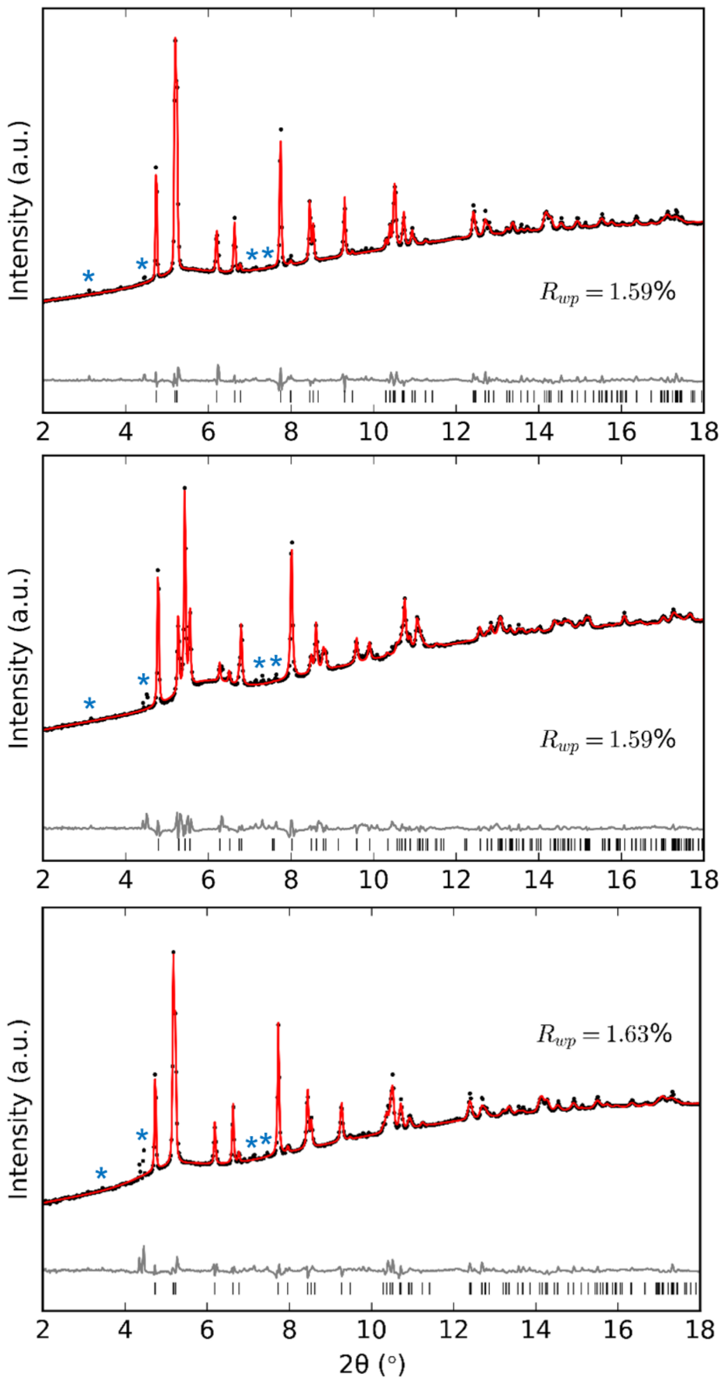

Figure S11: Powder X-ray diffraction patterns of $Cu(dca)_2$ phases: orthorhombic (top, 0.05 GPa), monoclinic (middle, 1.37 GPa), and orthorhombic after pressure-induced decomposition (bottom), measured at Diamond Light Source (I15) using radiation with a wavelength of 0.4246 Å. Patterns were analyzed using Rietveld refinement, with experimental data shown in black, calculated patterns in red, residuals in grey, and permitted reflections indicated by vertical bars. Some unindexed reflections present in all patterns are attributed to hydrated $Cu(dca)_2$ impurities remaining after the vacuum drying process and are marked with blue asterisks; these reflections were not included in the refinement model.

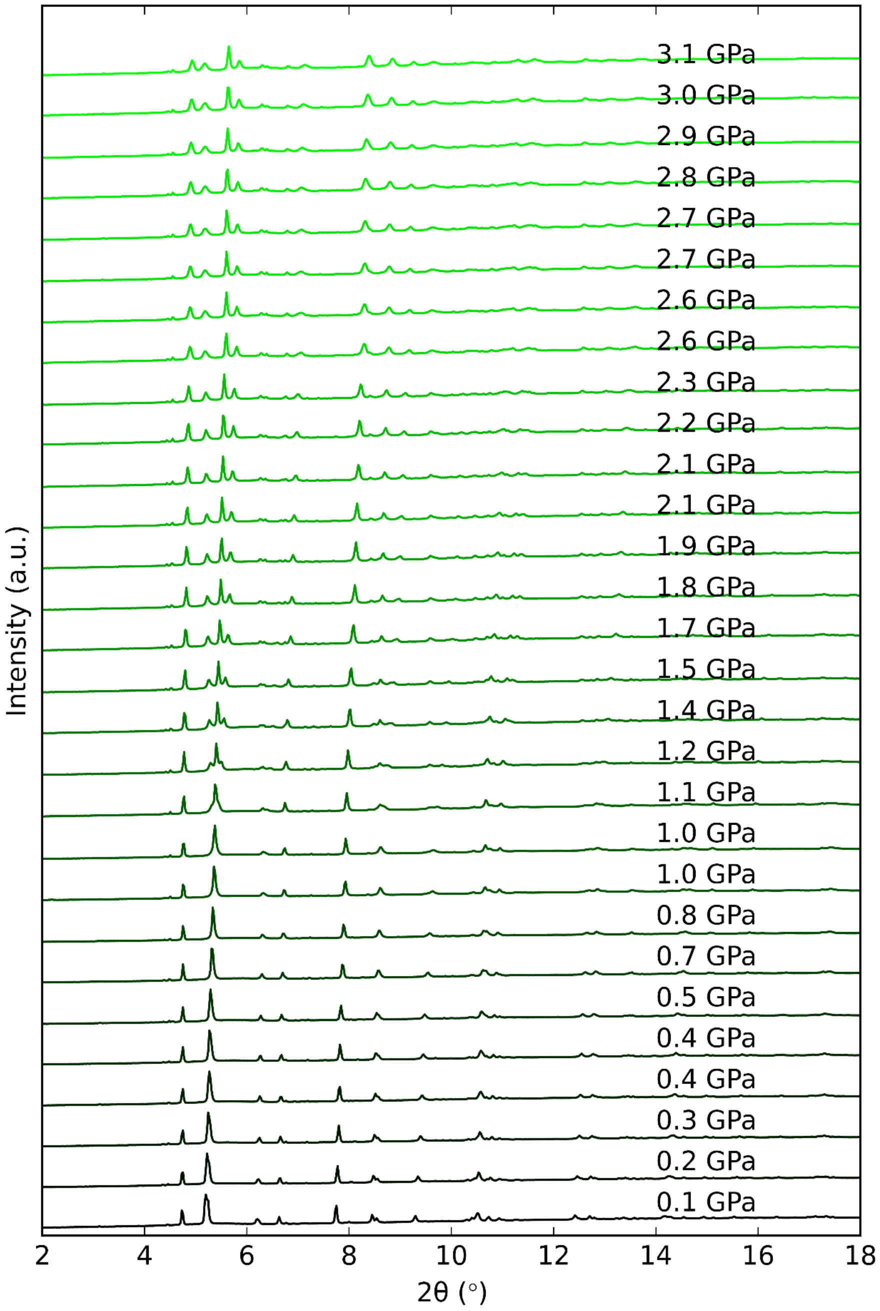

Figure S12: HP-PXRD patterns of $Cu(dca)_2$ measured at Diamond Light Source (X3) using radiation with a wavelength of 0.4246 Å. The patterns represent, in ascending order, the orthorhombic (0.1-1.1 GPa) and monoclinic (1.2-3.1 GPa) phases.

Table S6: Lattice parameters and unit cell volumes of $Cu(dca)_2$ as a function of pressure during compression, except for the last point which was collected after decompression. Pressure uncertainties are estimated at ±0.1 GPa or less, with smaller uncertainties for fine pressure steps.

p / GPa	Space group	*a* / Å	*b* / Å	*c* / Å	β / °	*V* / Å^3	R_{wp}
0.05	*Pnnm*	6.0900(7)	7.3367(8)	7.1855(12)	90	321.05(7)	1.59
0.10	*Pnnm*	6.0689(8)	7.3360(9)	7.1913(12)	90	320.17(8)	1.52
0.16	*Pnnm*	6.0435(11)	7.3236(10)	7.1890(14)	90	318.19(9)	1.38
0.27	*Pnnm*	6.0024(13)	7.312(2)	7.194(2)	90	315.74(12)	1.33
0.36	*Pnnm*	5.9785(13)	7.3018(15)	7.193(2)	90	314.02(13)	1.31
0.43	*Pnnm*	5.962(2)	7.296(2)	7.198(2)	90	313.1(2)	1.33
0.51	*Pnnm*	5.938(2)	7.285(2)	7.199(3)	90	311.4(2)	1.39
0.59	*Pnnm*	5.913(2)	7.272(2)	7.198(3)	90	309.5(2)	1.39
0.69	*Pnnm*	5.886(2)	7.260(2)	7.201(3)	90	307.7(2)	1.52
0.80	*Pnnm*	5.859(2)	7.247(2)	7.211(3)	90	306.2(2)	1.60
0.88	*Pnnm*	5.839(2)	7.238(2)	7.218(4)	90	305.0(2)	1.72
0.96	*Pnnm*	5.822(2)	7.228(2)	7.221(5)	90	303.8(2)	1.89
1.03	*Pnnm*	5.814(2)	7.223(3)	7.232(6)	90	303.7(3)	2.11
1.11	$P2_1/c$	5.7849(11)	7.2032(7)	7.213(3)	91.370(13)	300.46(13)	1.61
1.24	$P2_1/c$	5.7598(10)	7.1797(11)	7.211(2)	92.205(11)	298.00(10)	1.72
1.37	$P2_1/c$	5.7453(8)	7.1564(8)	7.226(2)	93.001(10)	296.68(8)	1.59
1.50	$P2_1/c$	5.7332(8)	7.1358(8)	7.231(2)	93.511(10)	295.25(8)	1.51
1.58	$P2_1/c$	5.7201(8)	7.1168(7)	7.2310(14)	93.859(9)	293.70(7)	1.46
1.69	$P2_1/c$	5.7084(7)	7.0935(7)	7.2323(13)	94.254(9)	292.05(7)	1.43
1.82	$P2_1/c$	5.6944(7)	7.0642(7)	7.2324(13)	94.657(8)	289.97(7)	1.35
1.90	$P2_1/c$	5.6851(7)	7.0440(7)	7.2309(13)	94.922(9)	288.50(10)	1.36
2.15	$P2_1/c$	5.6778(7)	7.0220(8)	7.2307(14)	95.190(9)	287.10(7)	1.36
2.12	$P2_1/c$	5.6686(8)	6.9910(9)	7.229(2)	95.515(9)	285.17(8)	1.39
2.19	$P2_1/c$	5.6631(9)	6.9693(10)	7.228(2)	95.726(10)	283.85(9)	1.42
2.29	$P2_1/c$	5.6614(10)	6.9489(11)	7.232(2)	95.933(11)	282.97(10)	1.48
2.59	$P2_1/c$	5.6356(13)	6.8849(15)	7.228(3)	96.563(15)	278.63(13)	1.70
2.63	$P2_1/c$	5.6343(13)	6.880(2)	7.229(3)	96.62(2)	278.36(14)	1.71
2.66	$P2_1/c$	5.6333(14)	6.878(2)	7.232(3)	96.68(2)	278.29(15)	1.74
2.71	$P2_1/c$	5.6303(15)	6.871(2)	7.231(3)	96.73(2)	277.8(2)	1.73
2.78	$P2_1/c$	5.623(2)	6.863(2)	7.230(3)	96.83(2)	277.0(2)	1.73
2.89	$P2_1/c$	5.616(2)	6.845(2)	7.224(3)	96.93(2)	275.7(2)	1.67
2.96	$P2_1/c$	5.612(2)	6.816(2)	7.218(3)	97.11(2)	274.0(2)	1.61
3.06	$P2_1/c$	5.613(2)	6.790(2)	7.215(3)	97.27(2)	272.8(2)	1.57
0	*Pnnm*	6.1168(9)	7.3481(8)	7.190(12)	90	323.18(8)	1.63

5. Variable-pressure neutron diffraction data of $Cu(tcm)_2$

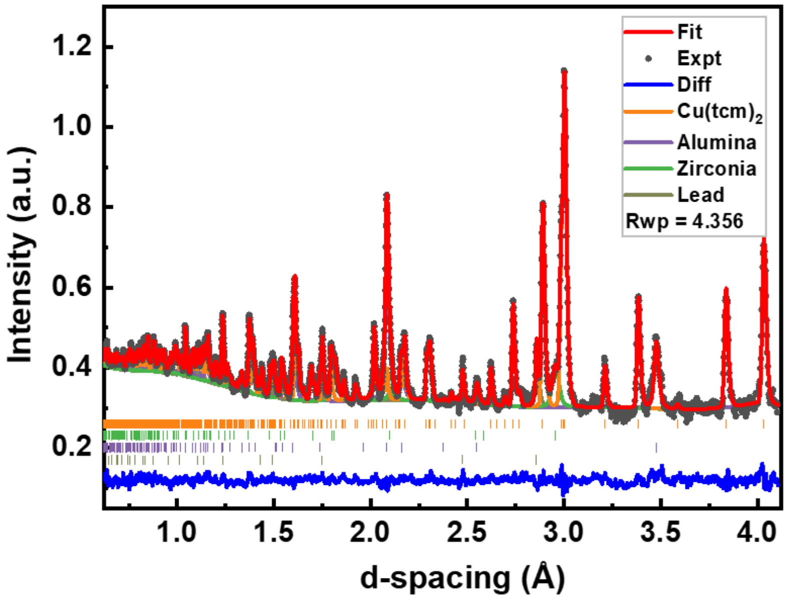

Figure S13: Neutron diffraction pattern of $Cu(tcm)_2$ at 0.11 GPa, fitted using Rietveld refinement. The experimental data are displayed in black, the fit in red, and the residuals in blue. The calculated signals for alumina, zirconia, and lead, which come from anvil and pressure marker, are represented by purple, green, and dark green lines, respectively, while the permitted reflections are represented by vertical bars. The R_{wp} value is given as a satisfactory indicator of refinement.

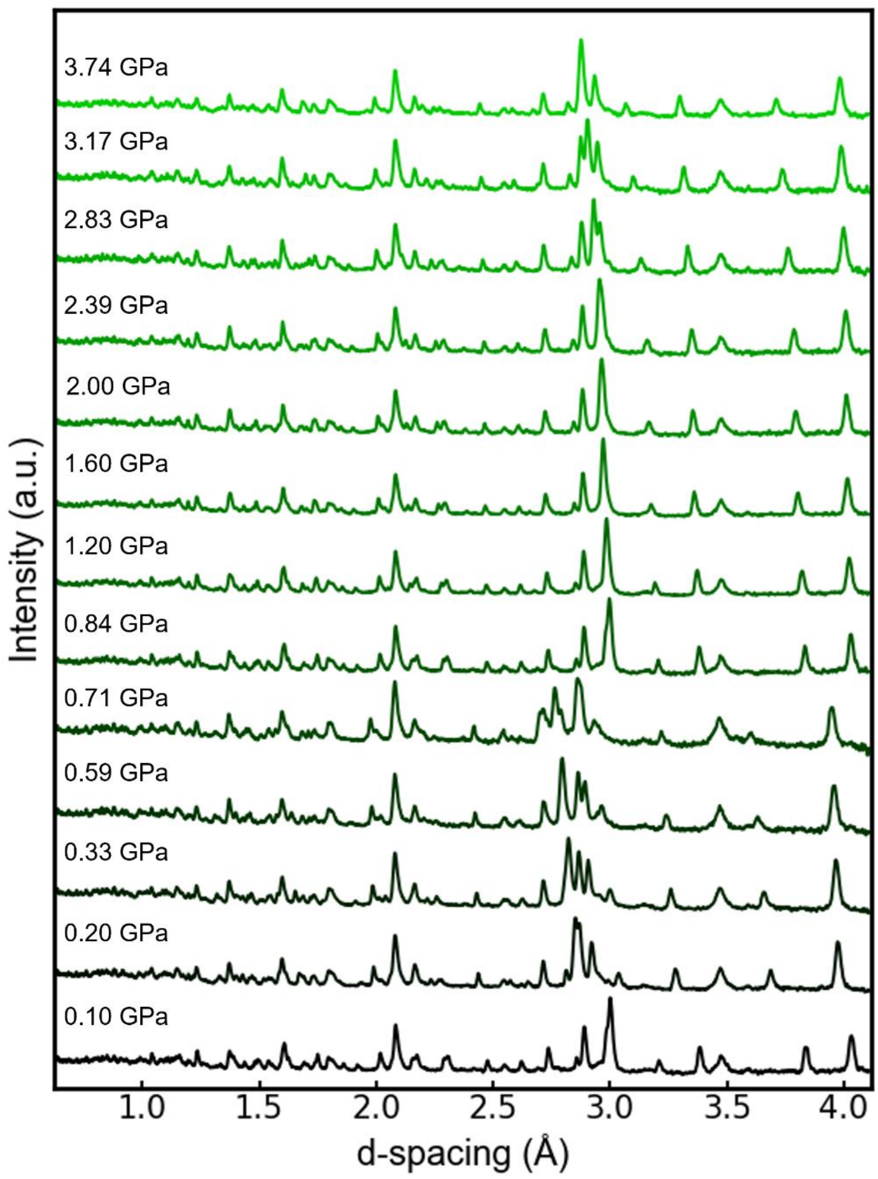

Figure S14: High-pressure neutron diffraction patterns of $Cu(tcm)_2$ measured at ISIS Neutron and Muon Source. The patterns represent the orthorhombic phase of $Cu(tcm)_2$ over the entire pressure range.

Table S7: Lattice parameters of $Cu(tcm)_2$ as a function of pressure during compression.

p / GPa	Space group	*a* / Å	*b* / Å	*c* / Å	β / °	*V* / Å^3	R_{wp}
0.0106(9)	*Pmna*	7.1693(2)	5.4692(2)	10.7653(2)	90	422.11(2)	4.36
0.203(10)	*Pmna*	7.1685(2)	5.4665(2)	10.7481(3)	90	421.18(2)	4.39
0.339(11)	*Pmna*	7.1708(2)	5.4563(2)	10.6950(2)	90	418.46(2)	3.95
0.597(10)	*Pmna*	7.1724(2)	5.4450(2)	10.6210(2)	90	414.79(2)	3.84
0.713(11)	*Pmna*	7.1721(2)	5.4405(2)	10.5847(3)	90	413.02(2)	4.47
0.843(11)	*Pmna*	7.1732(2)	5.4370(2)	10.5496(3)	90	411.44(2)	4.28
1.198(12)	*Pmna*	7.1752(2)	5.4278(2)	10.4346(2)	90	406.38(2)	4.50
1.613(13)	*Pmna*	7.1745(2)	5.4231(2)	10.3064(2)	90	401.00(2)	4.88
2.00(2)	*Pmna*	7.1759(3)	5.4232(2)	10.1762(3)	90	396.02(3)	4.35
2.39(2)	*Pmna*	7.1762(2)	5.4243(2)	10.0471(2)	90	391.10(2)	4.39
2.83(2)	*Pmna*	7.1752(2)	5.4261(2)	9.9045(3)	90	385.61(2)	4.33
3.17(3)	*Pmna*	7.1745(3)	5.4287(2)	9.7668(4)	90	380.40(3)	5.09
3.75(4)	*Pmna*	7.1895(6)	5.4177(4)	9.6312(7)	90	375.14(5)	7.53

6. Variable-pressure X-ray diffraction data of $Cu(tcm)_2$

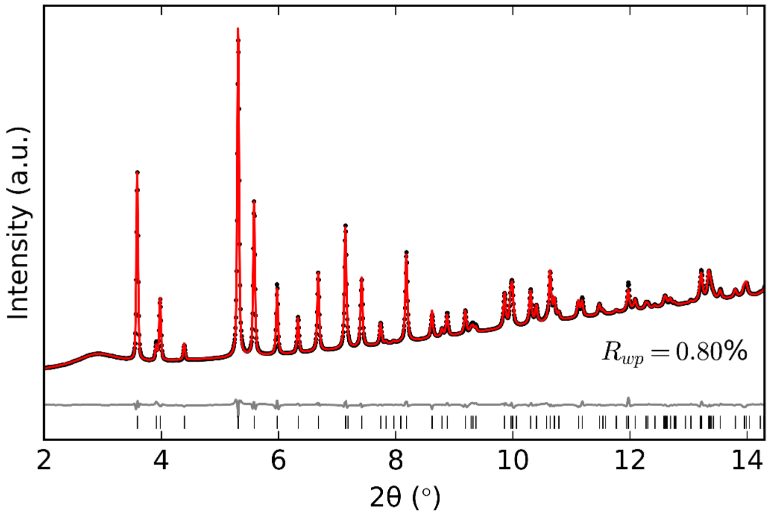

Figure S15: Powder X-ray diffraction patterns of the orthorhombic phases of $Cu(tcm)_2$ at 0.02 GPa, measured at the ESRF using radiation with a wavelength of 0.3738 Å. It was fitted using Rietveld refinement. The experimental data are displayed in black, the fit in red, the residuals in grey, and the permitted reflections are denoted by vertical bars.

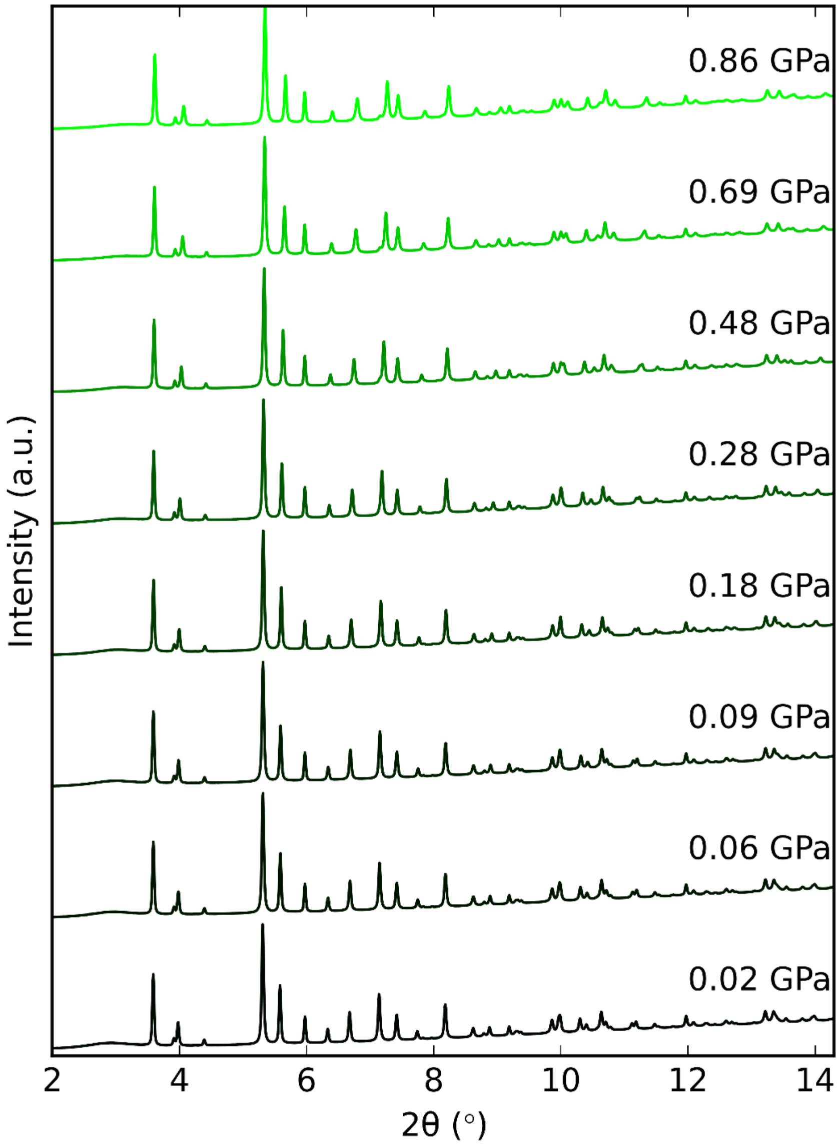

Figure S16: HP-PXRD patterns of $Cu(tcm)_2$ measured at the ESRF using radiation with a wavelength of 0.3738 Å. The patterns represent the orthorhombic phase of $Cu(tcm)_2$ during compression.

Table S8: Lattice parameters and unit cell volumes of $Cu(tcm)_2$ as a function of pressure during compression. Pressure uncertainties are estimated at ±0.1 GPa or less, with smaller uncertainties for fine pressure steps.

p / GPa	Space group	*a* / Å	*b* / Å	*c* / Å	*β* / °	*V* / Å^3	R_{wp}
0.02	*Pmna*	7.16771(12)	5.46999(11)	10.7605(2)	90	421.892(14)	0.80
0.06	*Pmna*	7.16831(12)	5.46760(11)	10.7478(2)	90	421.243(14)	0.80
0.09	*Pmna*	7.16857(13)	5.46552(11)	10.7367(2)	90	420.665(14)	0.81
0.18	*Pmna*	7.16910(12)	5.46068(11)	10.7107(2)	90	419.303(14)	0.82
0.28	*Pmna*	7.16946(13)	5.45530(12)	10.6795(2)	90	417.708(15)	0.86
0.48	*Pmna*	7.17061(14)	5.44675(12)	10.6224(2)	90	414.876(15)	0.85
0.69	*Pmna*	7.1724 (2)	5.43905(15)	10.5628(3)	90	412.07(2)	0.94
0.86	*Pmna*	7.1732(2)	5.4346(2)	10.5184(4)	90	410.05(2)	1.04

7. References

1 R. J. Angel, M. Alvaro and J. Gonzalez-Platas, *Z. für Krist. - Cryst. Mater.*, 2014, **229**, 405–419.

2 R. J. Angel, *Reviews in Mineralogy and Geochemistry*, 2000, **41**, 35–59.

3 H. M. Rietveld, *Acta Crystallogr.*, 1967, **22**, 151–152.

4 A. A. Coelho, *J. Appl. Crystallogr.*, 2018, **51**, 210–218.

5 S. R. Batten, R. Robson, P. Jensen, B. Moubaraki and K. S. Murray, *Chem. Commun.*, 1998, **0**, 439–440.

6 S. R. Batten, B. F. Hoskins, B. Moubaraki, K. S. Murray and R. Robson, *J. Chem. Soc., Dalton Trans.*, 1999, **0**, 2977–2986.

7 A. A. Yakovenko, K. W. Chapman and G. J. Halder, *Acta Crystallogr. Sect. B*, 2015, **71**, 252–257.

8 M. J. Cliffe and A. L. Goodwin, *J. Appl. Crystallogr.*, 2012, **45**, 1321–1329.
